# Genome-wide identification of leaf abscission associated microRNAs in sugarcane (*Saccharum officinarum L.*)

**DOI:** 10.1186/s12864-017-4053-3

**Published:** 2017-09-25

**Authors:** Ming Li, Zhaoxu Liang, Shanshan He, Yuan Zeng, Yan Jing, Weikuan Fang, Kaichao Wu, Guanyu Wang, Xia Ning, Lunwang Wang, Song Li, Hongwei Tan, Fang Tan

**Affiliations:** 1 Sugarcane Research Institute, Guangxi Academy of Agricultural Sciences, Nanning, 530007 People’s Republic of China; 2 Guangxi Academy of Agricultural Sciences, Nanning, 530007 People’s Republic of China

**Keywords:** Sugarcane-NGS-smallRNA-leaf absicission associated miRNA

## Abstract

**Background:**

Sugarcane (*Saccharum officinarum L*.) is an economically important crop, mainly due to the production of sugar and biofuel (Azevedo RA, Carvalho RF, Cia MC, & Gratão PL, Trop Plant Biol 4:42-51, 2011). Grown mainly in tropical and subtropical countries, sugarcane is a highly polyploid plant with up to ten copies of each chromosome, which increases the difficulties of genome assembly and genetic, physiologic and biochemical analyses. The increasing demands of sugar and the increasing cost of sugarcane harvest require sugarcane varieties which can shed their leaves during the maturity time, so it is important to study the mechanism of leaf abscission in sugarcane.

**Results:**

To improve the understanding of miRNA roles in sugarcane leaf abscission, we reported the genome-wide characterization of miRNAs and their putative targets in sugarcane using deep sequencing for six small RNA libraries. In total, 93 conserved miRNAs and 454 novel miRNAs were identified in sugarcane using previously reported transcriptome as reference. Among them, 25 up-regulated and 13 down-regulated miRNAs were identified in leaf abscission sugarcane plants (LASP) compared to leaf packaging sugarcane plants (LPSP). Target prediction revealed several miRNA-mRNA modules including miR156-SPL, miR319-TPR2, miR396-GRF and miR408-LAC3 might be involved in the sugarcane leaf abscission. KEGG pathway enrichment analysis showed differentially expressed miRNAs may regulate pathways like “plant hormone signal transduction” and “plant-pathogen interaction”, which is consistent with previous transcriptome study. In addition, we identified 96 variant miRNAs with 135 single nucleotide polymorphisms (SNPs). The expression of sugarcane miRNAs and variant miRNAs were confirmed by qRT-PCR. We identified a possible poaceae specific miRNA called miR5384 for the first time in sugarcane.

**Conclusions:**

We not only reported miR5384, a possible poaceae specific miRNA, for the first time in sugarcane but also presented some miRNA-mRNA modules including miR156-SPL, miR319-TPR2, miR396-GRF and miR408-LAC in sugarcane. These modules might be involved in the regulation of sugarcane leaf abscission during the maturity time. All of these findings may lay ground work for future application of sugarcane breeding program and benefit research studies of sugarcane miRNAs.

**Electronic supplementary material:**

The online version of this article (doi:10.1186/s12864-017-4053-3) contains supplementary material, which is available to authorized users.

## Background

Abscission is the programmed developmental process that some of the organs such as leaves, flowers, or fruits are shed during the life of a plant [1], which can be divided into four major steps: (i) development of the abscission zone (AZ) tissue, (ii) acquisition of competence to respond to abscission-promoting signaling, (iii) activation of abscission and (iv) post abscission trans-differentiation [2]. Global gene expression studies have shown many genes in multiple pathways participate in the abscission process [3, 4], including various transcription factors (TFs), cell wall hydrolysis enzymes, defense-related genes and genes involved in auxin/ethylene signal transduction [5–7]. However, the regulation mechanism of gene expression is not clear at present.

MicroRNAs (miRNAs) are endogenous small (21–24 nt) and single stranded noncoding RNAs which are important regulators of gene expression through mRNA degradation, translational repression and chromatin modification [8–10]. They are incorporated in the RNA-induced silencing complex (RISC) with AGO protein and guide the cleavage or translational repression of the target mRNAs by complementary or uncomplimentary base pairing [11]. In plants, miRNAs are involved in multiple crucial biological processes including organ development and plant responses to environmental stresses [12–16]. Notably, several miRNAs have been reported to be involved in the abscission process. miR159 targeting MYB transcription factors, miR160/miR167 targeting auxin response factors (ARFs), miR172 targeting AP2-like ethylene-responsive transcription factors, and miR396 targeting glutamate decarboxylase have been found with different expression in tomato during pedicel abscission [17]. In fruit senescence of *Fragaria ananassa*, NAC TFs, ARFs and MYB TFs have been validated as targets of miR164, miR160, miR167 and miR159, respectively, by small RNA sequencing and degradome sequencing [18].

Sugarcane (*Saccharum officinarum L*.) is an economically important crop, mainly due to the production of sugar and biofuel [19]. Grown mainly in tropical and subtropical countries, sugarcane is a highly polyploid plant with up to ten copies of each chromosome, which increases the difficulties of genome assembly and genetic, physiologic and biochemical analyses. The increasing demands of sugar and the increasing cost of sugarcane harvest require sugarcane varieties which can shed their leaves during the maturity time, so it is important to study the mechanism of leaf abscission in sugarcane.

Recently, we have reported leaf abscission associated genes in sugarcane using transcriptome sequencing [20]. To study miRNA expression changes between leaf abscission sugarcane plants (LASP) and leaf packaging sugarcane plants (LPSP), we constructed six small RNA libraries and sequenced them using an Illumina HiSeq 2500 system. In total, we characterized 93 conserved miRNAs and 454 sugarcane novel miRNAs using the transcriptome sequences as reference and identified 38 differentially expressed miRNAs in LASP compared to LPSP. Target prediction showed several miRNA-mRNA modules might be involved in sugarcane leaf abscission, such as miR156-SPL, miR319-TPR2, miR396-GRF and miR408-LAC3. KEGG pathway analysis for the target genes showed similar results as our transcriptome study and indicated “plant-pathogen interaction” and “plant hormone signal transduction” might be related with sugarcane leaf abscission during the maturity time. We obtained highly conserved sugarcane pre-miRNAs and mature miRNAs, which are valuable resources for future sugarcane miRNA studies. Moreover, our findings will provide better understanding of the complex mechanism of leaf abscission, miRNAs and their targets involved in leaf abscission.

## Results

### Small RNA identification in sugarcane

**Fig. 1:**
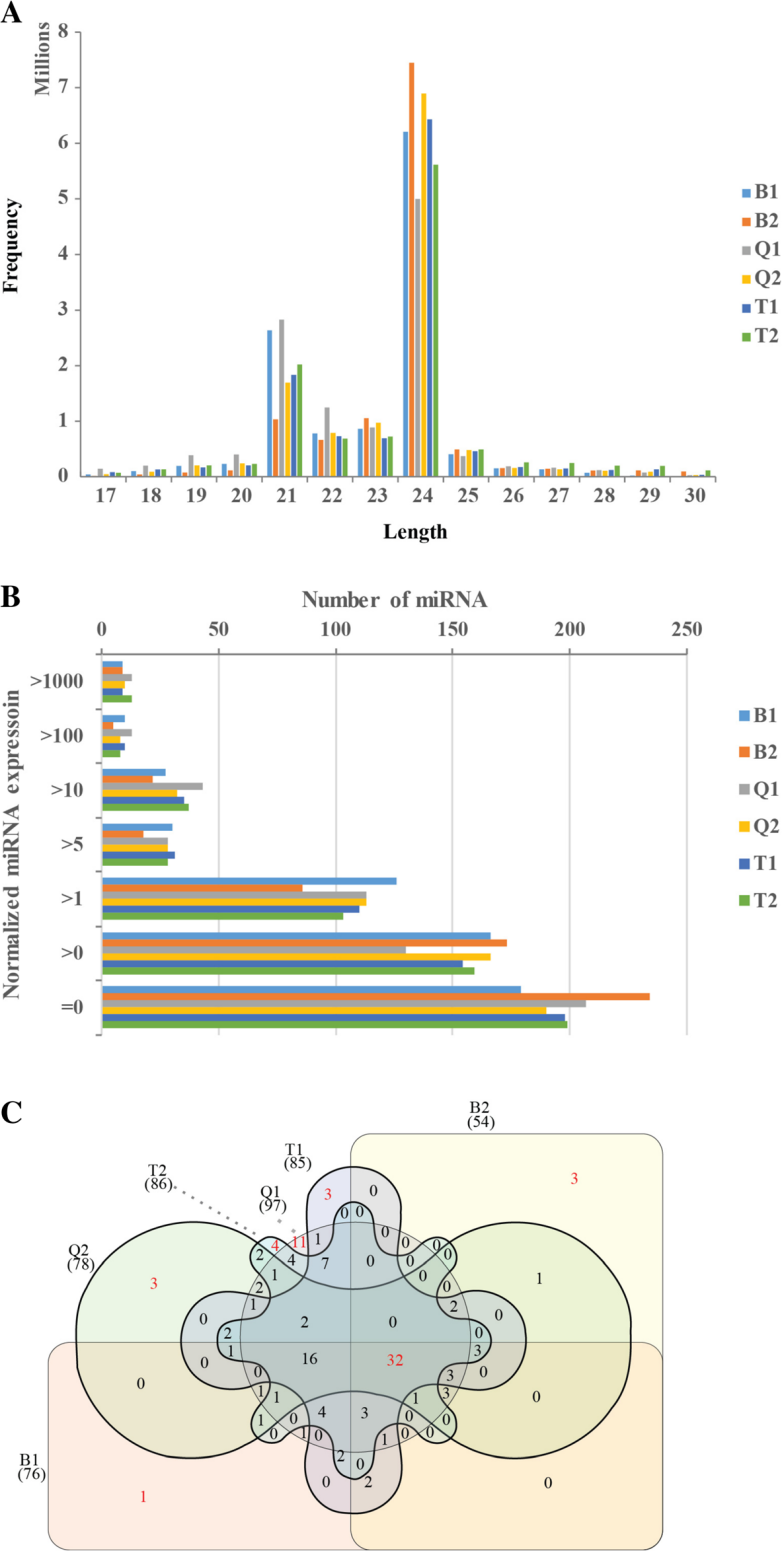
Overview of small RNA sequencing. **a** Length distribution showed small RNA reads peaked at 21 nt and 24 nt. **b** Plot of numbers of miRNAs with different expression levels in each sample showed approximate 71.47% ~ 82.75% of the total detected miRNAs were expressed no more than 5 TPM (excluding miRNA with 0 TPM) in the samples. **c** Venn diagram of miRNAs (> 1 TPM) showed 32 miRNAs were commonly detected in all six samples

Previously, we reported leaf abscission associated genes in sugarcane using transcriptome sequencing [20]. To study the post-transcriptional regulation of sugarcane leaf abscission, six small RNA (sRNA) libraries constructed for three leaf abscission sugarcane plants (Q1, T1, T2) and three leaf packaging sugarcane plants (Q2, B1, B2) were sequenced by using an Illumina HiSeq 2500 system. Initially, a total of 71,579,415 raw reads were generated (Table 1). After low quality reads and sequencing adapters were removed, we obtained 70,082,453 clean reads longer than 18 nt for all samples with an average of 11.68 M clean reads. Length distribution of clean reads (Fig. 1a) showed the most abundant classes of sRNA were 21 and 24 nt, which is consistent with many plant miRNA deep sequencing studies [21–23]. To annotate sRNAs in each library, we mapped the clean reads to Rfam database and found 5.10–10.93% of the clean reads were derived from rRNA, tRNA, snRNA and snoRNA (Table 1). Next, all clean reads were aligned to recently published sugarcane transcriptome [20] for global miRNA characterization. Mapped reads were used to predict sugarcane miRNAs (pre-miRNAs and mature miRNAs) by MIREAP [24] and a total of 93 conserved miRNAs (including mature and passenger miRNAs) from 25 families and 454 sugarcane novel miRNAs were identified (Additional file 1: Supplementary Dataset). These miRNAs were used as references for sRNA mapping, miRNA expression profiling and SNP scanning. Notably, a possible poaceae specific miRNA miR5384, which has been identified only in *Sorghum bicolor* and *Triticum aestivum* in miRBase, is reported for the first time in sugarcane.

**Table 1:** Summary of small RNA sequencing and annotation in LASP and LPSP

	Q1	Q2	T1	T2	B1	B2
Total reads	12,265,513	12,244,515	11,581,880	11,470,897	12,089,516	11,927,094
Clean reads	11,932,261	11,966,866	11,323,908	11,243,443	11,841,904	11,774,071
Mapping to the reference	6,289,451	4,794,303	4,752,371	5,508,483	5,463,043	4,142,894
sRNA reads mapping to miRNAs	1,323,246	992,446	1,171,729	1,404,371	1,895,595	594,461
Known miRNA	81	85	84	85	84	69
Novel miRNA	259	272	265	263	284	244
rRNA, tRNA, snRNA, snoRNA, etc.	1,304,588	695,355	723,361	1,027,076	711,685	601,042
unannotated sRNA reads	9,304,427	10,279,133	9,428,818	8,811,996	9,234,624	10,578,568

### miRNA expression profile

We next profiled sugarcane miRNA expression in each sample by mapping the clean reads to sugarcane miRNA precursors using BLAST software [25]. sRNA reads matched to sugarcane miRNAs without mismatches were counted. To compare miRNA expression in different libraries, in this study the number of clean reads was used as background for normalization and TPM (transcripts per million reads) was used to present the expression levels of miRNAs. A total of 439 miRNAs were detected ((Additional file 2: Table S1) and 340, 357, 349, 348, 368 and 313 miRNAs were detected more than 1 TPM in Q1, Q2, T1, T2, B1and B2, respectively (Table 1). Distribution of normalized miRNA expression (Fig. 1b) revealed approximate 71.47% ~ 82.75% of the total detected miRNAs were expressed no more than 5 TPM (excluding miRNA with 0 TPM), and 26 (7.65%), 18 (5.04%), 19 (5.44%), 21 (6.03%), 19 (5.16%) and 14 (4.47%) miRNAs were identified >100 TPM in Q1, Q2, T1, T2, B1 and B2, respectively. For subsequent analyses, miRNAs whose normalized expression values were lower than 5 TPM in all samples were removed. Venn diagram (Fig. 1c) revealed 32 miRNAs were common to all six samples, and 11, 3, 3, 4, 1 and 3 miRNAs were detected more than 5 TPM only in Q1, Q2, T1, T2, B1 and B2, respectively.

### Analysis of differentially expressed miRNAs

**Fig. 2:**
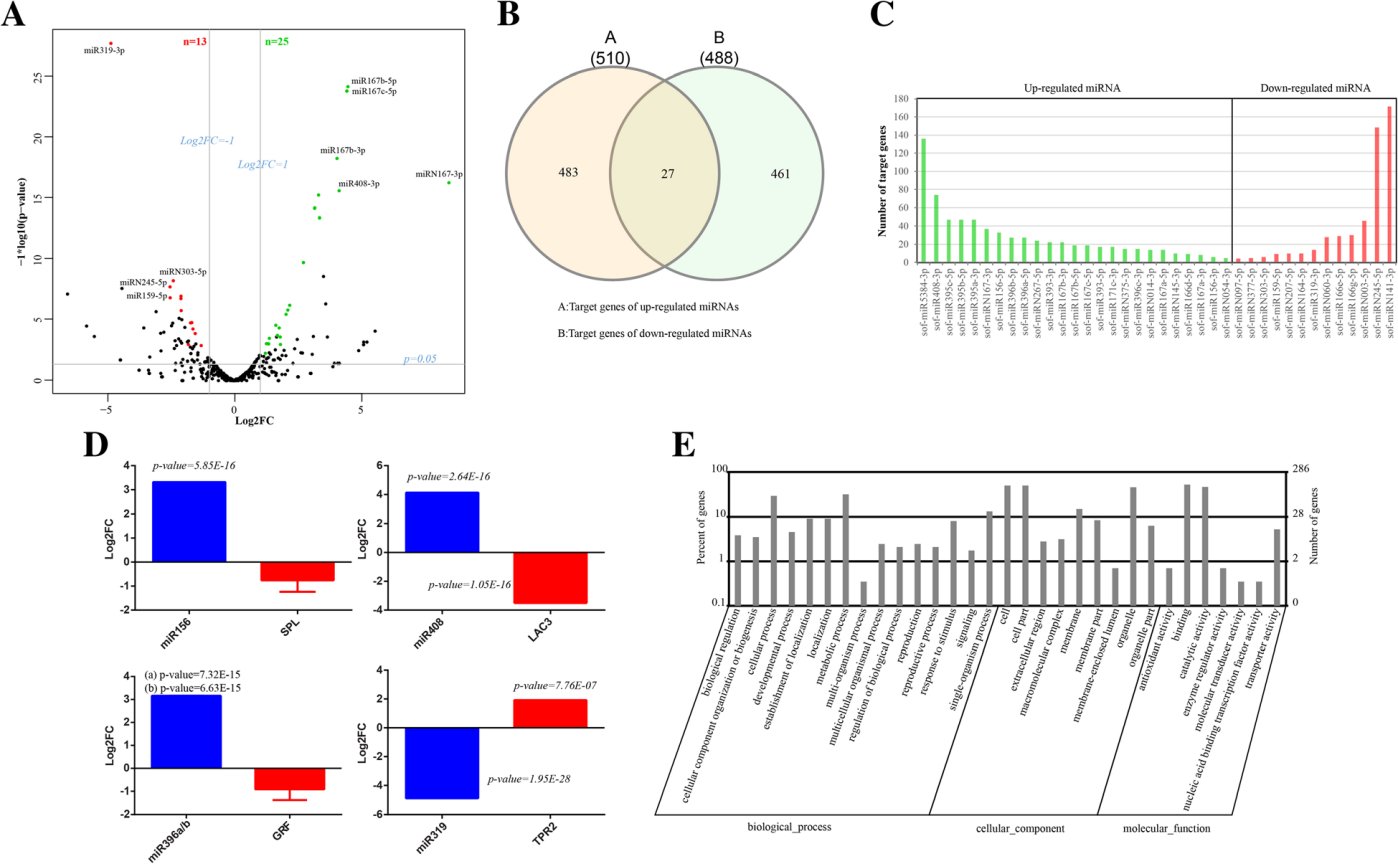
Differentially expressed miRNAs between LASP and LPSP. **a** Volcano plot showed 25 up-regulated miRNAs (in *green*) and 13 down-regulated (in *red*) were identified in LASP compared to LPSP. **b** Venn diagram showed up- and down-regulated miRNAs can target 510 and 488 target genes, of which 27 were shared. **c** Number of target genes regulated by up- and down-regulated miRNAs. **d** Log2FCs of four miRNAs (miR156, miR319, miR396 and miR408) and their target genes (SPL, GRF, TPR2 and LAC3) showed not only all of them were differentially expressed significantly (*p* < 0.05) but also these miRNAs may regulate the expression of their targets. **e** Gene ontology analysis of differentially expressed miRNAs

In general, miRNAs differentially expressed in LASP and LPSP are associated with leaf abscission process in sugarcane. We next identified differentially expressed miRNAs between LASP and LPSP using edgeR [26]. In this study, we used several statistical values as cut-offs: normalized expression > 5 TPM, log2 fold change (Log2FC) > 1 (up-regulated) or Log2FC <− 1(down-regulated), *p*-value < 0.05 and FDR < 0.05. With this criterial we obtained 25 up-regulated and 13 down-regulated miRNAs in LASP compared to LPSP (Fig. 2a, Table 2). It is clear that some miRNAs were differentially expressed significantly in LASP and LPSP because their statistical values were far away from the log2 fold change and FDR cut-offs, including sugarcane known miRNAs (miR167b-5p, miR167b-3p, miR167c-5p, miR408-3p, miR319-3p and miR159-5p) and novel miRNAs (miRN167-3p, miRN245-5p and miRN303-5p). It is interesting that both mature (also known as functional strand) and passenger (also known as the miRNA* strand) miRNAs from MIR167 family were up-regulated in LASP. Passenger miRNAs are usually thought to be degraded quickly during the miRNA biogenesis but they still can be active in silencing [27]. Like *Arabidopsis thaliana* and *Oryza sativa*, passenger miRNAs from MIR167 family are derived from the 3′-arms of the pre-miRNAs in sugarcane. Sugarcane passenger miRNA of MIR167 (miR167-3p) has been reported with high abundance in sugarcane root and predicted to be functional in plant development [28]. This is the first time to report leaf abscission associated miRNAs in sugarcane. A total of four pairs of mature and passenger miRNAs were differentially expressed between LASP and LPSP and they were derived from miRNA families like MIR156, MIR167 and MIR393 (Table 2).

**Table 2:** Differentially expressed miRNAs between LASP and LPSP

miRNA	LPSP^a^			LASP^a^			Log2FC^b^	p-value^c^	FDR^d^
	B1	B2	Q2	Q1	T1	T2			
sof-miRN167-3p	0	0	0	14.331	0.177	0	8.451	5.50E-17	5.01E-15
sof-miR167b-5p	23.223	0.595	11.448	807.81	63.317	135.635	4.471	6.69E-25	1.83E-22
sof-miR167c-5p	24.996	1.444	9.526	822.728	64.377	130.387	4.433	1.50E-24	2.15E-22
sof-miR167a-5p	24.996	1.444	9.526	821.722	64.2	129.853	4.430	1.57E-24	2.15E-22
sof-miR408-3p	0.422	0.17	0.836	18.186	5.387	11.562	4.126	2.64E-16	2.06E-14
sof-miR167b-3p	2.027	0.085	1.421	21.79	6.535	43.759	4.042	5.59E-19	6.12E-17
sof-miR167a-3p	3.378	1.019	0.251	30.673	6.358	28.372	3.355	4.40E-14	2.19E-12
sof-miR156-5p	34.454	15.712	60.25	1288.775	76.917	230.445	3.317	5.85E-16	4.00E-14
sof-miR396b-5p	654.118	30.491	108.299	6328.725	927.242	1603.601	3.166	6.63E-15	4.01E-13
sof-miR396a-5p	655.3	30.491	108.968	6310.874	927.153	1603.868	3.160	7.32E-15	4.01E-13
sof-miR396c-3p	3.124	0	1.17	25.812	4.857	6.848	2.721	2.06E-10	8.68E-09
sof-miRN375-3p	3.124	0	1.17	25.812	4.857	6.848	2.721	2.06E-10	8.68E-09
sof-miR395c-5p	1.942	0.934	1.671	5.615	9.184	12.541	2.178	6.80E-07	1.62E-05
sof-miR395a-3p	1.942	1.104	1.671	5.615	9.184	12.541	2.106	1.68E-06	3.84E-05
sof-miR395b-5p	1.942	1.104	1.671	5.447	8.036	12.541	2.036	3.71E-06	7.51E-05
sof-miR156-3p	0.338	0.51	0.585	5.783	1.06	0.889	1.816	0.001132	0.009671
sof-miRN145-3p	1.351	0.595	0.084	0.084	9.449	0.089	1.807	0.000279	0.003154
sof-miRN267-5p	2.364	0	0.752	7.878	2.561	3.38	1.765	4.95E-05	0.000714
sof-miR5384-3p	1.52	0	0.836	9.638	0	1.067	1.693	0.000205	0.002547
sof-miRN054-3p	19	14.014	8.022	115.904	19.516	63.771	1.634	3.06E-05	0.000507
sof-miR171c-3p	25.165	0.679	2.507	80.119	6.8	8.005	1.378	0.000357	0.003909
sof-miR166d-5p	4.222	1.274	3.51	20.533	3.797	7.649	1.352	0.001059	0.009198
sof-miR393-3p	9.289	3.142	5.933	30.17	10.509	21.079	1.306	0.001004	0.009006
sof-miR393-5p	45.516	11.975	24.651	150.433	31.261	83.426	1.261	0.000992	0.009006
sof-miRN014-3p	0	0.425	3.844	6.788	6.711	0.356	1.219	0.006096	0.040176
sof-miRN164-5p	14.187	7.134	8.189	0	5.652	9.872	−1.307	0.001391	0.011189
sof-miRN060-3p	8.445	9.682	8.858	5.531	4.592	4.091	−1.543	0.000147	0.001867
sof-miRN141-3p	33.272	1.529	18.551	0	0	18.411	−1.631	5.92E-05	0.00083
sof-miRN097-5p	45.094	23.866	23.147	24.639	7.948	10.05	−1.678	1.79E-05	0.000326
sof-miR166e-5p	31.583	5.606	17.465	1.257	7.241	10.94	−1.728	1.86E-05	0.000328
sof-miRN003-5p	5.236	0.085	0.501	0.251	0.177	1.512	−1.814	0.001153	0.009704
sof-miRN377-5p	1.013	10.277	0.836	1.341	2.384	1.779	−2.094	1.86E-06	4.08E-05
sof-miR166g-5p	56.325	17.241	59.498	2.179	9.537	25.437	−2.103	1.23E-07	3.53E-06
sof-miR159-5p	7.6	14.863	24.651	10.392	0.442	6.226	−2.106	1.93E-07	5.02E-06
sof-miRN207-5p	7.093	15.797	15.961	5.28	3.444	3.113	−2.406	6.38E-09	2.33E-07
sof-miRN245-5p	0.084	7.134	1.086	0.503	0.883	1.334	−2.529	1.64E-07	4.50E-06
sof-miRN303-5p	1.013	11.975	0.084	0.587	2.384	1.512	−2.534	2.08E-08	7.10E-07
sof-miR319-3p	404.58	1660.938	5.766	163.842	1.325	1.512	−4.864	1.95E-28	1.07E-25

^a^Normalized miRNA expression, > 5 in one of the six samples

^b^Log 2 fold change, > 1 or <− 1

^c^P-value calculated by edgeR, < 0.05

^d^False discovery rate, < 0.05

### Target prediction for differentially expressed miRNAs

The identification of miRNA targets and their regulation is a crucial step to understand the biological functions of miRNAs. Using the assembled sugarcane transcriptome we computationally predicted the target genes for up- and down-regulated miRNAs using the method recommended by Allen [29] and Schwab [30]. As shown in Fig. 2b, we predicted 510 and 488 target genes for up-regulated and down-regulated miRNAs, respectively (Additional file 1: Supplementary Dataset), and there were 27 target genes shared by up- and down-regulated miRNAs. It is revealed that all differentially expressed miRNAs between LASP and LPSP were predicted to have target genes in sugarcane leaves (Fig. 2c). Among up- and down-regulated miRNAs, miR5384-3p and miRN141-3p were predicted regulate most target genes, respectively (Fig. 2c).

Annotation of miRNA target genes (Additional file 1: Supplementary Dataset) revealed several conserved miRNA-mRNA modules identified, including miR156-SPL (squamosa promoter-binding-like protein), miR319-TPR (topless-related protein), miR396-GRF (growth-regulating factor) and miR408-LAC3 (laccase-3). In this study, miR156 was predicted to target 15 SPL genes (e.g. SPL3, SPL11, SPL12, SPL14, SPL17 and SPL19), miR396a/b targeting 8 GRF genes (e.g. GRF3, GRF4, GRF9 and GRF12) and miR408 targeting one LAC3 gene (laccase-3). As shown in Fig. 2d, using both sRNA and transcriptome [20] data we showed the expression changes of normalized expression values of miRNAs and mRNAs from these modules in LASP and LPSP. It is interesting that all these three miRNAs (miR156, miR396 and miR408) were up-regulated while their target genes (SPL, GRF and LAC3) were down-regulated significantly (*p*-value < 0.05) in LASP compared to LPSP, which increased the reliability of the regulation of miRNA-mRNA in sugarcane leaf abscission. In addition, miR319-3p was down-regulated in LASP compared to LPSP, but its putative target gene TPR2 (topless-related protein 2) was up-regulated significantly (Fig. 2d). We also found opposite expression patterns of novel miRNAs and their target genes (Additional file 1: Supplementary Dataset), such as miRN245-RGA2 (disease resistance protein RGA2) and miRN375-GRF (growth-regulating factor).

### Functional analysis of differentially expressed miRNAs

Function analysis for miRNA target genes showed differentially expressed miRNAs were involved in multiple pathways (Fig. 2e and Table 3). Gene Ontology analysis (Fig. 2d) revealed differentially expressed miRNAs between LASP and LPSP may function in “cellular process” (GO:0009987), “metabolic process” (GO:0008152), “response to stimulus” (GO:0050896), “cell” (GO:0044464), “membrane” (GO:0016020), “binding” (GO:0005488) and “catalytic activity” (GO:0003824) while KEGG pathway enrichment analysis (Table 3) revealed 5 significant pathways (*p*-value < 0.05 and q-value < 0.05) were regulated by differentially expressed miRNAs between LASP and LPSP. It is notable that 22 genes that were targets of 5 differentially expressed miRNAs (miR171c-3p, miR393-5p, miR5384-3p, miR167a-3p and miRN141-3p) were involved in the pathway of “plant hormone signal transduction” (ko04075) and both miR393-5p and miR393-3p were involved in the same pathway - “Glycosylphosphatidylinositol(GPI)-anchor biosynthesis” (ko00563). Two cell growth and death pathways were found to be regulated by two miRNAs (miRN245-5p and miR5384-3p). Detailed information of KEGG pathway enrichment analysis can be accessed in Additional file 2: Table S2. We found other not significant pathways regulated by differentially expressed miRNAs maybe important for leaf abscission in sugarcane as well. For example, “flavonoid biosynthesis” (ko00941) regulated by miR5384-3p, “apoptosis” (ko04210) regulated by miR5384-3p and miR166e-5p, and “plant-pathogen interaction” (ko04626) regulated by 11 miRNAs (miR166e-5p, miR167b-5p, miR393-3p, miR159-5p, miRN014-3p, miRN141-3p, miR396b-5p, miR396a-5p, miR167c-5p, miR167a-5p and miRN245-5p). In our transcriptome study [20], “plant-pathogen interaction” was the most significant pathway involved by differentially expressed genes. In current study, 20 genes from this pathway were predicted to be regulated by 11 miRNAs mentioned before, which indicated that “plant-pathogen interaction” may be important for sugarcane leaf abscission. Due to the limit annotations of the assembled transcriptome, functions of many target genes of differentially expressed miRNAs are currently unknown.

**Table 3:** KEGG pathway analysis for the target genes of differentially expressed miRNAs

Group	Pathway	ID	Target_gene	p-value^a^	q-value^b^	miRNA
Digestive system	Mineral absorption	ko04978	8	3.32E-08	5.08E-06	miRN141-3p, miRN167-3p
Glycan biosynthesis and metabolism	Glycosylphosphatidylinositol(GPI)-anchor biosynthesis	ko00563	9	7.80E-06	0.0012	miR393-3p, miR393-5p
Cell growth and death	Meiosis - yeast	ko04113	9	2.10E-05	0.0032	miRN245-5p, miR5384-3p
Signal transduction	Plant hormone signal transduction	ko04075	22	0.00022	0.03335	miR171c-3p, miR393-5p, miR5384-3p, miR167a-3p, miRN141-3p
Cell growth and death	Oocyte meiosis	ko04114	9	0.0003	0.04587	miR5384-3p, miRN245-5p

^a^p-value calculated by student’s t-test, < 0.05

^b^q-value calculated by an R package called “qvalue”, < 0.05

### miRNA SNPs

**Fig. 3:**
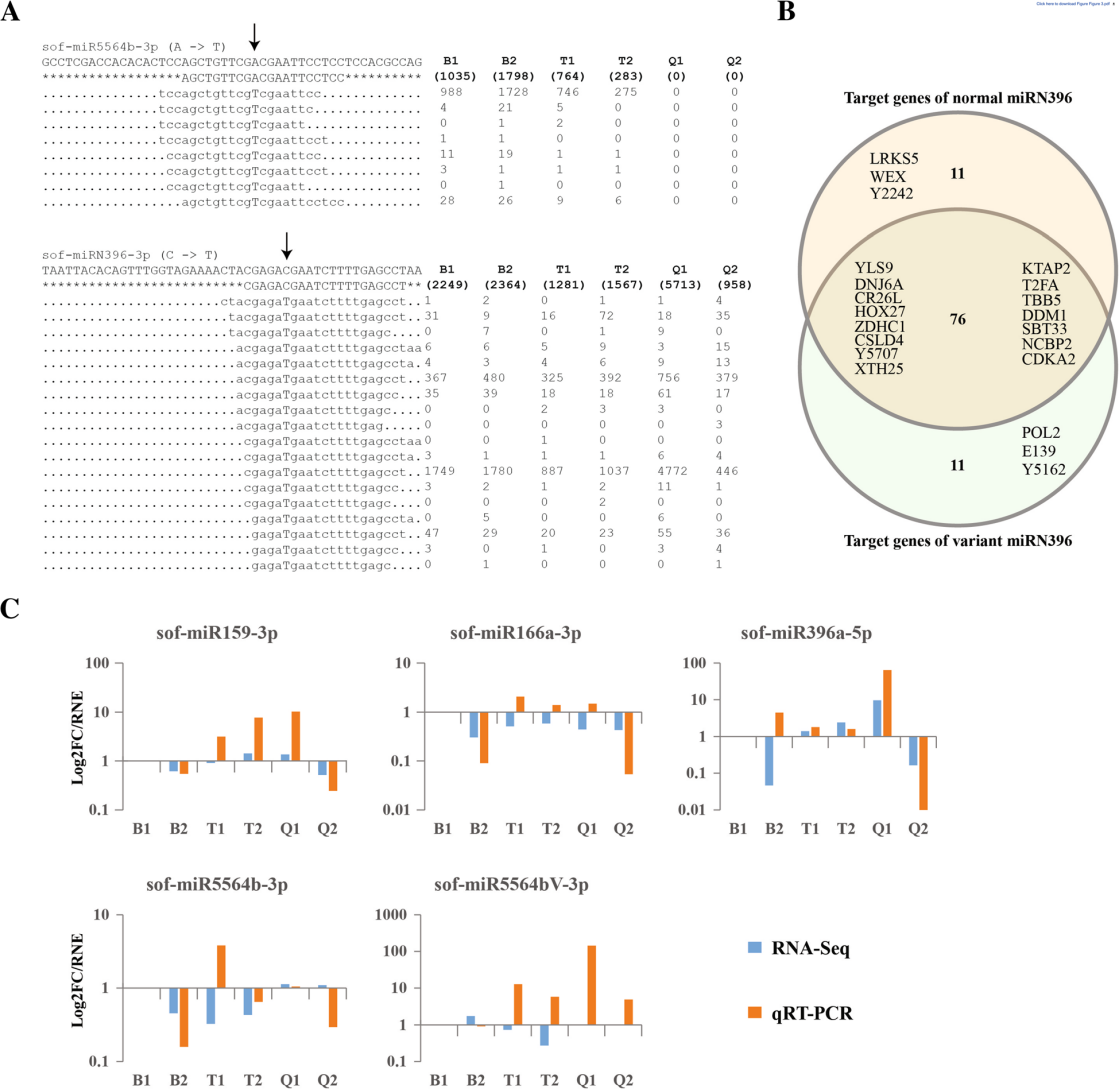
SNPs in sugarcane miRNAs and qRT-PCR validation. **a** Reads mapping of variant sof-miR5564b-3p and sof-miRN396-3p showed SNPs in their sequences. **b** Venn diagram of target genes of normal and variant sof-miRN396 showed because of the SNP (C - > T) in the “seed” region sof-miRN396 lost 11 target genes (e.g. LRKS5, WEX, Y2242) but gained new target genes (POL2, E139, Y5162). **c** qRT-PCR validation for candidate miRNAs (sof-miR159-3p, sof-miR166a-3p, sof-miR396a-5p and sof-miR5564b-3p) and variant miRNA (sof-miR5564bV-3p)

The biogenesis of miRNA, guide miRNA selection and miRNA-mRNA interaction indicate that single nucleotide polymorphisms (SNPs) in miRNA genes or mature miRNA should affect the biogenesis and function of miRNAs [31]. It is implicated that sugarcane leaf abscission may be related to them, so we next scanned SNPs in sugarcane miRNAs in all samples. After variant miRNAs with low frequency (< 10 reads) and small rate (< 1%) were removed, we obtained 135 SNPs happened in 96 sugarcane miRNAs (Additional file 2: Table S3). Among these variant miRNAs two isoforms (sof-miR5564b-3p and sof-miRN396-3p) attracted our attention because of their high expression in the samples (Fig. 3a). An “A to T” and a “C to T” happened to sof-miR5564b-3p and sof-miRN396-3p, respectively. It is interesting that variant miRNAs were differentially expressed between LASP and LPSP. Variant sof-miR5564b-3p was expressed much higher in LPSP (B1 and B2) compared to LASP (T1 and T2), but there was no evidence of it in Q1 and Q2. In this study we identified two main variant isoforms of sof-miRN396-3p – “acgagaTgaatcttttgagcct” and “cgagaTgaatcttttgagcct”. They were found with high sequence similarity with normal sof-miRN396-3p (cgagaCgaatcttttgagcct). In addition, variant sof-miRN396-3p was expressed higher than its normal form in most samples. The numbers of reads mapping to normal sof-miRN396-3p were 1305, 1197, 1318, 1565, 1908 and 1092 reads in B1, B2, Q1, Q2, T1 and T2, respectively, but variant sof-miRN396-3p was aligned with 2249, 2364, 5713, 958, 1281 and 1567 reads in B1, B2, Q1, Q2, T1 and T2, respectively (Fig. 3a). In plants most miRNAs cleave their downstream targets depending on the highly complementary recognition sites [32]. SNPs in miRNA regions, especially in their “seed” regions (2nd–7th nucleotides of the miRNA), has huge influence on miRNA biogenesis and function, such as miRNA guide strand selection and downstream target gene binding [27, 33]. We predicted the target genes using the transcriptome reference for normal and variant sof-miRN396-3p (Fig. 3b). Although normal and variant sof-miRN396-3p shared 76 target genes such as YLS9 (YELLOW-LEAF-SPECIFIC GENE 9), HOX27 (Homeobox-leucine zipper protein), DDM1 (ATP-dependent DNA helicase) and CDKA2 (Cyclin-dependent kinase A-2), variant sof-miRN396-3p losses the regulation of 11 genes (e.g. LRKS5 (L-type lectin-domain containing receptor kinase S.5), WEX (Werner Syndrome-like exonuclease), Y2242 (LRR receptor-like serine/threonine-protein kinase)) and gains11 new genes (e.g. POL2 (Retrovirus-related Pol polyprotein from transposon 297), E139 (Glucan endo-1,3-beta-glucosidase 9), Y5162 (Uncharacterized protein At5g41620)) due to the SNP (C - > T) in its “seed” region.

### Stem-loop qRT-PCR

To validate the expression of normal miRNAs and variant miRNAs in LASP and LPSP, we performed stem-loop qRT-PCR experiment. Four miRNAs (sof-miR159-3p, sof-miR166a-3p, sof-miR396a-5p and sof-miR5564b-3p) and one variant miRNA (sof-miR5564bV-3p) were selected as candidates and actin was used as an internal control. Reverse transcriptase, forward and reverse primers were designed and synthesized in BGI-Shenzhen (Additional file 2: Table S4). After ΔCt value of each miRNA candidate was calculated in each sample, the expression of miRNAs was normalized using B1 as the control (Fig. 3b). A total of 13 out of 20 (65.5%) were identified with same expression patterns in all samples between small RNA-Seq and qRT-PCR, which support that miRNA expression profile by small RNA sequencing is reliable. To validate the variant isoform of sof-miR5564, we used two different forward primers with one nucleic acid difference and the results showed it was detectable in most of the samples. The overall correlation of normal and variant miRNAs between qRT-PCR and deep sequencing, calculated as 0.274, indicated that miRNAs identified by deep sequencing were accuracy and effective, and can be used for miRNA expression profiles analysis of sugarcane leaf abscission during maturity time.

## Discussion

**Fig. 4:**
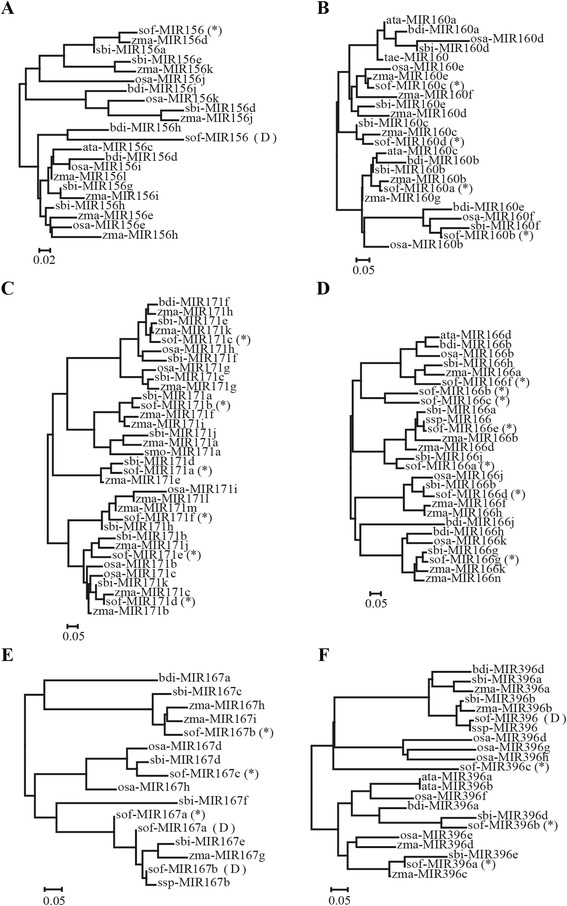
Polygenetic trees of six miRNA families. Polygenetic trees of pre-miRNAs, including (**a**) MIR156, (**b**) MIR160, (**c**) MIR166, (**d**) MIR167, (**e**) MIR171 and (**f**) MIR396, identified in this study and miRBase (v 21). MiRNAs with “(D)” are marked because they are sugarcane miRNAs recorded in current miRBase

In this study, we characterized 93 conserved miRNAs from 25 families and 454 novel miRNAs in sugarcane using deep sequencing technology. Unlike previous sugarcane miRNA studies [34–39], this is the first time to identify sugarcane miRNAs using the sugarcane transcriptome reference. Using sugarcane transcriptome reference, we not only characterized pre-miRNAs and mature miRNAs in sugarcane but also analyze miRNA-mRNA interactions. Pre-miRNAs identified in this study are more reliable because they have high similarities with other species. Polygenetic trees (Fig. 4 a ~ f) of six miRNA families (MIR156, MIR160, MIR166, MIR167, MIR171 and MIR396) performed by MEGA 7 [40] revealed pre-miRNAs identified in this study were conserved across species, such as *Sorghum bicolor* (sbi), *Zea mays* (zma), *Oryza sativa* (osa) and other monocotyledons. In addition, multiple sequence alignment (Additional file 2: Figure S1A-F) performed by ClustalX (v 2.0) [41] showed mature miRNAs, step-loop regions and passenger miRNAs were conserved across species, unlike previously reported sugarcane miRNAs (marked with “D”). Considering this we submitted these sugarcane miRNAs to miRBase for updates.

Leaf abscission is a complicated process regulated by developmental, hormonal and environmental cues [42]. In other plants multiple types of genes have been reported to relate with leaf abscission, such as auxin response proteins, ethylene-synthesis enzymes, cell wall-degrading enzymes, pathogenesis related proteins [43, 44]. In this study four miRNAs (miR156, miR319, miR396 and miR408) and their target genes (SPL, TPR2, GRF and LAC3) were identified with different expression trends (up-regulation or down-regulation) in LASP and LPSP (Fig. 2d). SPLs are trans-acting factors that bind specifically to the consensus nucleotide sequence (5′-TNCGTACAA-3′) of AP1 promoter and can promote both vegetative phase change (e.g. leaf epidermal differentiation) and flowering time [45–47]. There are 16, 18, 13, and 31 SPL genes in *Arabidopsis thaliana*, *Oryza sativa*, *Physcomitrella patens*, and *Zea mays*, respectively, most of which can be regulated by miR15648 [48]. In Arabidopsis, overexpression of SPL3 that is a consequence of miR156 decrease can accelerate the production of abaxial trichome and short petioles of adult leaves [45]. Another study also has confirmed the regulation of miR156 in SPLs and has revealed the decrease of miR156 and overexpression of SPLs (SPL3, SPL9 and SPL10) produce less lateral roots in Arabidopsis [49]. In rice SPLs and miR156 have been reported to be involved in multiple developmental processes, especially the flower development [50]. In our transcriptome study we have identified several SPL genes down-regulated in LASP compared to LPSP [20], the up-regulation of miR156 in current study strongly support that the module miR156-SPL may function in sugarcane leaf abscission.

In this study, we also found the up-regulation of miR396 and miR408 in LASP compare to LPSP. miR396 is a highly conserved miRNA (Fig. 4f) and always functions, through repressing GRFs (growth-regulating factors), in the regulation of developmental processes of leaf, flower and other organs in plants [51–54]. Kim and Lee have conducted an experiment to show that GRF4 mutation results in small leaves of Arabidopsis [52]. Not only GRF4 but also other GRF genes have been confirmed to be regulated by miR396 and overexpression of miR396 results reduction of GRFs and narrow-leaf phenotypes in Arabidopsis [53]. In other plants such as tobacco and citrus miR396 has also been reported to target GRFs and regulate the development of leaf, flower and fruit [54, 55]. In addition, it has been experimented that overexpression of miR396 can reduce the ability of salt and alkali stress tolerance in rice and Arabidopsis [56]. Unlike miR396, the overexpression of miR408 can increase drought tolerance by targeting different genes in plants, such as rice [57], chickpea [58] and *Medicago truncatula* [59]. In Arabidopsis miR408 is increased while its target LAC3 is decreased during senescence [60]. Similarly, current study showed correlated expression patterns of miR396-GRF and miR408-LAC3 in LASP and LPSP.

By regulating MYB and TCP transcription factors, miR319 is involved in various developmental processes such as leaf development and senescence, organ curvature and hormone biosynthesis and signaling [61–63]. In this study we found miR319 can target MYB transcription factor but the expression of MYB was not significantly changed in LASP and LPSP (Additional file 1: Supplementary Dataset). However, TPR2 (topless-related protein 2) was predicted to be regulated by miR319 and its expression was correlated with miR319 (Fig. 2d). TPR2 is a transcriptional corepressor involved in the regulation of branch formation, strigolactones signaling and auxin signaling [64, 65]. At the early leaf development stage of Arabidopsis TIE1 (TCP Interactor containing EAR motif protein 1) can regulate leaf size by inhibiting TCP activities through recruiting the TOPLESS (TPL)/TOPLESS-RELATED (TPR) corepressors [66]. The down-regulation of miR319 and up-regulation of its target TPR2 in LASP compared to LPSP indicate the module of miR319-TPR2 might be involved in sugarcane leaf abscission. Due to the limit annotations for miRNA target genes, it is hard to understand very well about the functions of all differentially expressed miRNAs between LASP and LPSP. Further experiments should be conducted to explore the functions of differentially expressed miRNAs and their targets in sugarcane leaf abscission.

## Conclusions

In conclusion, we successfully identified 547 miRNAs in sugarcane, of which 25 were up-regulated and 13 were down-regulated in LASP compared to LPSP. We not only reported miR5384, a possible poaceae specific miRNA, for the first time in sugarcane but also presented some miRNA-mRNA modules including miR156-SPL, miR319-TPR2, miR396-GRF and miR408-LAC in sugarcane. These modules might be involved in the regulation of sugarcane leaf abscission during the maturity time. All of these findings may lay ground work for future application of sugarcane breeding program and benefit research studies of sugarcane miRNAs.

## Methods

### Plant material and growth conditions

New born leafs from six sugarcane cultivars (Q1, Q2, T1, T2, B1 and B2) with different phenotypes on leaf abscission during the maturity time, as described [20]. Briefly, Q2 (ROC-26) is a precocious and productive sugarcane variety from Taiwan, but the sugar cane is wrapped tightly at the physiological maturity [67]. Q1 (GT96–167) is a late-maturing and high-yield sugarcane variety which is bred by Guangxi Sugarcane Research Institute [68–70]. In contrast with Q2, Q1 can shed their leaves easily during the maturity time. T1, T2, B1 and B2 are four generation varieties of Q1 and Q2. T1 and T2 can shed their leaves as well during the maturity time, rather than B1 or B2. All the sugarcane varieties were proved to have stable agronomic characteristic on leaf shedding by 5 years of filed observation. All six sugarcane plants were planted in January of 2014 in the experimental field of Sugarcane Research Institute in Nanning, Guangxi Province of China. In December of 2014, after the sugarcane plants were validated in maturity time according to the sugar assay test, new born leaf tissues approximately 5 cm above the growing point were taken and stored in the liquid nitrogen before RNA extraction.

### Total RNA extraction

Leaf tissues (100 mg) of Q1, Q2, T1, T2, B1 and B2 were used to isolate total RNA by using TRIzol® reagent (Invitrogen) according to the manufacture’s protocol [71]. The quality of total RNA was controlled by Agilent 2100 Bioanalyzer.

### Small RNA library construction and deep sequencing

Six small RNA libraries (Q1, Q2, T1, T2, B1 and B2) were constructed and sequenced with Illumina TruSeq deep sequencing technology (Sample Preparation Guide, Par #15004197 Rev.A, Illumina, San Diego, CA). Briefly, small RNAs (18–30 nt) were size-selected by gel fraction and extracted by centrifugation. After ligation of 5′ and 3′ adaptors, small RNAs were reverse transcribed into cDNA, then amplified using the sequencing primers for 14 cycles and the fragments (~ 150 bps) were isolated from a 6% TBE PAGE-gel. After the cDNA was purified, it was used for cluster generation and sequenced using an Illumina HiSeq 2000 platform. Image files generated by the sequencer were processed to nucleotide sequences (raw FASTQ files) using a base-calling pipeline (Illumina). FASTQ files for all six libraries have been submitted to Sequence Read Archive (SRA) of NCBI under the accession number SRA347706 (Q1: SRR3184695, Q2: SRR3184696, T2: SRR3184697, T1: SRR3184698, B1: SRR3184699, and B2: SRR3184700).

### Reference transcriptome de novo assembly and annotation

Previously, we have demonstrated the transcriptome analysis for these six sugarcane varieties [20]. The raw sequencing data is available with the accession number SRA291189 from SRA platform. The assembled transcriptome was used as the reference for small RNA identifications in current study [72]. Transcriptome sequences were assembled by Trinity software [73]. Then, the assembled transcripts were annotated by mapping them to NCBI non-redundant (NR), UniProtKB/Swiss-Prot, Gene Ontology (GO) and Kyoto Encyclopedia of Genes and Genomes (KEGG) databases. The mapping hits were filtered using a cut-off of e-value (1 × 10^−5^), as previously described [20]. The assembled transcriptome sequences can be accessed in NCBI Transcriptome Shotgun Assembly (TSA) Database using the accession number GELY00000000.

### Identification of sugarcane pre-miRNAs and mature miRNAs

Raw small RNA reads were cleaned by removing the adaptors, short (< 18 nt) reads and low quality reads, resulting clean reads were used for sequential analysis. To identify sugarcane miRNA precursors, we mapped all clean reads to the assembled transcriptome reference using SOAP2 software [74] under maximum two mismatches. Then, MIREAP [24] was used for global miRNA search in the assembled transcriptome of sugarcane [20] with parameters as follows: minimal length, 18; maximal length, 25; maximal copy number of miRNAs on reference, 20; maximal free energy allowed for a miRNA precursor, − 18 kcal/mol; maximal space between mature miRNA and passenger miRNA, 300; minima space between mature miRNA and passenger miRNA, 16; maximal bugle of mature miRNA and passenger miRNA, 4; flank sequence length of miRNA precursor, 20. Predicted pre-miRNAs and mature miRNAs were filtered with the expression and structure features by MIREAP. Two pre-miRNAs overlapped more than 80% nucleotides were considered as one. The structures of pre-miRNAs were predicted using RNAfold from ViennaRNA Package 2.0 [75]. Next, predicted sugarcane mature miRNAs were compared to miRBase [76] (v21, http://www.mirbase.org) for conserved miRNA identification. Conserved miRNAs were selected according to their similarities (> 90%) and expression (> 10 reads). Apart of these conserved miRNAs, the left miRNAs were considered as sugarcane novel miRNAs. All the sugarcane miRNA precursor and mature sequences have been submitted to miRBase.

### miRNA profile

To profile miRNA expression in each sample, BLAST software [71] was used to align the clean reads to sugarcane pre-miRNAs in each sample under perfect match. For a particular miRNA, its expression was calculated by counting the reads which had an overlap more than 18 nt with the miRNA. Then, the miRNA expression was normalized to the total clean reads using TPM (transcripts per million reads) method:

1 $$ TPM=\frac{1000000^{\ast}\left( mapped reads\right)}{Total clean reads} $$

### Different expression of miRNAs

Differentially expressed miRNAs between LASP and LPSP were identified using edgeR [26]. We used a strict criterial for selecting miRNAs with differential expression: normalized expression > 5 TPM, Log2FC > 1 or Log2FC < − 1, *p*-value < 0.05 and FDR < 0.05.

### Target prediction and function annotation of miRNAs

The assembled sugarcane transcriptome was aligned against by the mature miRNA sequences for target prediction, according to the suggestions by Allen [29] and Schwab [30]. Here, the criteria used for miRNA target prediction were as follows: i) no more than three mismatches between the miRNA and the target (G-U bases count as 0.5 mismatches); ii) no more than two adjacent mismatches in the miRNA/target duplex; iii) no adjacent mismatches in in positions 2–12 of the miRNA/target duplex (5′ of the miRNA); iv) no mismatches in positions 10–11 of miRNA/target duplex; v) no more than 2.5 mismatches in positions 1–12 of the of the miRNA/target duplex (5′ of the miRNA); and vi) the Minimum Free Energy (MFE) of the miRNA/target duplex should be ≥75% of the MFE of the miRNA bound to its perfect complement. Target genes were then used for GO and KEGG pathway enrichment analysis. We used *p-value* (Fisher’s exact test) and *q-value* [77] to show the significance of enrichment and control the false discovery rate. Significant GO items and KEGG pathways should satisfy the critical of *p*-value < 0.05 and q-value < 0.05. Detected KEGG pathways related to animal or human GO items were filtered.

### Stem-loop RT-qPCR

To validate the expression patterns of miRNAs in sugarcane leaves, we selected four miRNAs (sof-miR159-3p, sof-miR166a-3p, sof-miR396a-5p and sof-5564b-3p) and one variant miRNA (sof-miR5564bV-3p) to perform stem-loop reverse transcription and quantitative real-time PCR (RT-qPCR), following the protocols [78]. Initially, total RNA (2 μg) isolated from six sugarcane leaf samples was added into dNTP (2 μl, 2.5 mM, Geneland), 10 × RT Buffer (2 μl, QIAGEN), RT primer mix (2 μl, 1 μM), RNase Inhibitor (1 μl, Promega) and Quantiscript® Reverse Transcriptase (1 μl, 10 U/μl, QIAGEN) and diluted with RNase-free water to reverse transcription mix (20 μl). The reverse transcription mix was then incubated at 16 °C for 30 min, 42 °C for 40 min and at 85 °C for 5 min to finish cDNA synthesis. Next, cDNA template (1 μl) was mixed with 2× SYBR Green PCR mix (8 μl, QIAGEN), forward primer (0.2 μl, 50 pM/μl), reverse primer (0.2 μl, 50 pM/μl) and RNase-free H_2_O (6.6 μl) to make the PCR reaction mix. RT primers for cDNA synthesize and forward and reverse primers for RT-qPCR were designed and synthesized in BGI-Shenzhen (Additional file 2: Table S4). We used actin as an internal control and performed three replicates for each miRNA in every sample. The PCR reaction was performed and analyzed by the ABI ViiA 7 Real Time PCR System. The RT-qPCR conditions were as follows: preheating for 2 min at 95 °C; and 50 cycles of 94 °C for 10 s, 56 °C for 10 min and 72 °C for 40 s. All PCR products were denatured at 95 °C and cooled to 65 °C, and the fluorescence signals were accumulated consistently from 65 °C to 95 °C as the temperature increased at 0.2 °C per second. The expression levels of miRNAs were evaluated by the comparative threshold cycle (Ct) values. Relatively normalized expression (RNE, −ΔΔCT method) was used to show the expression change of a transcript in two samples [79]. CT values greater than 35 were set to 35.

### SNPs in miRNA

To characterize SNPs occurred in mature miRNAs of sugarcane, clean reads were aligned to sugarcane miRNA precursors by using BLAST software [25] with maximum one mismatch. Then, reads with perfect match to the pre-miRNAs and outside mature miRNA regions were removed. The expression of variant miRNAs was counted like normal mature miRNAs. Variant miRNAs with low expression (< 10 reads) or small ratio (< 1%) of total reads (variant miRNA reads and normal miRNA reads) were filtered. miRNAs that has two or more SNPs were not considered (Additional file 3).

## Additional files

Additional file 1: Supplementary Dataset. Tables of Sugarcane conserved and novel miRNAs and Target prediction of differentially expressed miRNAs. (XLS 408 kb)

Additional file 2: Summary of supplementary tables and figures. (PDF 1132 kb)

Additional file 3: Tables of overlapping miRNAs in six samples. (XLSX 36 kb)

## Abbreviations

ABA: Abscisic acid
ABC: ATP-binding cassette
ARFs: Auxin response factors
AZ: Abscission zone
EC: Enzyme commission
GO: Gene ontology
KEGG: Kyoto Encyclopedia of Genes and Genomes
LASP: Leaf abscission sugarcane plants
LPSP: Leaf packaging sugarcane plants
ORF: Open reading frame
qRT-PCR: Quantitative real-time polymerase chain reaction
SNP: Single nucleotide polymorphism
SRA: Sequence read archive
TF: Transcription factor
TPM: Transcripts Per Kilobase Million
