# Supplementary material for: Genome-wide identification of leaf abscission associated microRNAs in sugarcane (Saccharum officinarum L.)
Source: BMC Genomics. 2017 Sep 25;18:754. doi: 10.1186/s12864-017-4053-3 (PMC5613641; doi:10.1186/s12864-017-4053-3)

Supplementary Information

Genome-wide identification of leaf abscission associated microRNAs in sugarcane (*Saccharum officinarum L.*)

Ming Li[1,†,*], Zhaoxu Liang[1,†], Shanshan He[1], Yuan Zeng[2], Yan Jing[1], Kaichao Wu[1], Guanyu Wang[2], Xia Ning[2], Zhanghong Mo[1], Lunwang Wang[1] and Song Li[1], Fang Tan[1,*]

[1]Sugarcane Research Institute, Guangxi Academy of Agricultural Sciences, Nanning 530007, P.R.China.

[2]Guangxi Academy of Agricultural Sciences, Nanning 530007, P.R.China.

[†]Equal contributors.

[*]Correspondence to mingli9606@163.com and tanfang64@163.com.

Table S1. Raw miRNA reads and normalized miRNA expression in all samples.

miRNA	Raw miRNA reads						Normalized miRNA expression					
	B1	B2	Q1	Q2	T1	T2	B1	B2	Q1	Q2	T1	T2
sof-miR156-3p	4	6	69	7	12	10	0.338	0.51	5.783	0.585	1.06	0.889
sof-miR156-5p	408	185	15378	721	871	2591	34.454	15.712	1288.8	60.25	76.917	230.45
sof-miR159-3p	36537	22568	50319	19246	32150	49688	3085.4	1916.8	4217.1	1608.3	2839.1	4419.3
sof-miR159-5p	90	175	124	295	5	70	7.6	14.863	10.392	24.651	0.442	6.226
sof-miR160a-3p	17	3	0	2	2	0	1.436	0.255	0	0.167	0.177	0
sof-miR160a-5p	49	22	31	72	55	85	4.138	1.869	2.598	6.017	4.857	7.56
sof-miR160b-3p	1	0	0	2	3	3	0.084	0	0	0.167	0.265	0.267
sof-miR160b-5p	32	3	7	1	25	21	2.702	0.255	0.587	0.084	2.208	1.868
sof-miR160c-3p	2	0	0	3	3	2	0.169	0	0	0.251	0.265	0.178
sof-miR160c-5p	49	21	30	72	59	87	4.138	1.784	2.514	6.017	5.21	7.738
sof-miR160d-5p	49	22	31	72	55	85	4.138	1.869	2.598	6.017	4.857	7.56
sof-miR162-3p	1855	594	2599	1345	2108	2881	156.65	50.45	217.81	112.39	186.16	256.24
sof-miR162-5p	30	0	4	8	18	12	2.533	0	0.335	0.669	1.59	1.067
sof-miR164a-3p	4	0	0	4	8	7	0.338	0	0	0.334	0.706	0.623
sof-miR164a-5p	7	5	39	9	14	25	0.591	0.425	3.268	0.752	1.236	2.224
sof-miR164b-3p	5	0	0	2	2	0	0.422	0	0	0.167	0.177	0
sof-miR164b-5p	1	3	14	0	2	1	0.084	0.255	1.173	0	0.177	0.089
sof-miR164c-3p	4	1	0	6	3	8	0.338	0.085	0	0.501	0.265	0.712
sof-miR164c-5p	7	6	39	10	14	26	0.591	0.51	3.268	0.836	1.236	2.312
sof-miR166a-3p	2E+06	465884	692073	673343	758826	864545	129817	39569	58000	56267	67011	76893
sof-miR166a-5p	47	0	0	20	12	48	3.969	0	0	1.671	1.06	4.269
sof-miR166b-3p	132756	29259	194712	167750	197451	288109	11211	2485	16318	14018	17437	25625
sof-miR166b-5p	225	23	163	276	330	496	19	1.953	13.66	23.064	29.142	44.115
sof-miR166c-3p	2E+06	480774	854415	694047	830614	917297	134226	40833	71605	57997	73350	81585
sof-miR166c-5p	129	57	136	141	418	192	10.894	4.841	11.398	11.783	36.913	17.077
sof-miR166d-3p	2E+06	474015	848551	689212	818285	909069	133587	40259	71114	57593	72262	80853
sof-miR166d-5p	50	15	245	42	43	86	4.222	1.274	20.533	3.51	3.797	7.649
sof-miR166e-3p	101487	22073	172991	146777	185199	265240	8570.2	1874.7	14498	12265	16355	23591
sof-miR166e-5p	374	66	15	209	82	123	31.583	5.606	1.257	17.465	7.241	10.94
sof-miR166f-3p	2E+06	486004	862069	815802	938150	1E+06	137889	41277	72247	68172	82847	100119
sof-miR166f-5p	167	76	309	222	185	262	14.102	6.455	25.896	18.551	16.337	23.302
sof-miR166g-3p	39271	12773	23964	32295	18069	22675	3316.3	1084.8	2008.3	2698.7	1595.7	2016.7
sof-miR166g-5p	667	203	26	712	108	286	56.325	17.241	2.179	59.498	9.537	25.437
sof-miR167a-3p	40	12	366	3	72	319	3.378	1.019	30.673	0.251	6.358	28.372
sof-miR167a-5p	296	17	9805	114	727	1460	24.996	1.444	821.72	9.526	64.2	129.85
sof-miR167b-3p	24	1	260	17	74	492	2.027	0.085	21.79	1.421	6.535	43.759
sof-miR167b-5p	275	7	9639	137	717	1525	23.223	0.595	807.81	11.448	63.317	135.64
sof-miR167c-3p	0	0	10	0	6	0	0	0	0.838	0	0.53	0
sof-miR167c-5p	296	17	9817	114	729	1466	24.996	1.444	822.73	9.526	64.377	130.39
sof-miR168-3p	6770	3379	14332	5669	6468	13564	571.7	286.99	1201.1	473.73	571.18	1206.4
sof-miR168-5p	38703	9023	40431	22487	22193	44919	3268.3	766.35	3388.4	1879.1	1959.8	3995.1
sof-miR169a-3p	0	0	5	0	0	1	0	0	0.419	0	0	0.089
sof-miR169a-5p	1	1	16	0	0	0	0.084	0.085	1.341	0	0	0
sof-miR169b-5p	0	0	7	0	0	0	0	0	0.587	0	0	0
sof-miR171a-3p	62	10	71	53	61	167	5.236	0.849	5.95	4.429	5.387	14.853
sof-miR171a-5p	139	25	13	225	151	125	11.738	2.123	1.089	18.802	13.335	11.118
sof-miR171b-3p	61	10	69	52	60	166	5.151	0.849	5.783	4.345	5.299	14.764
sof-miR171c-3p	298	8	956	30	77	90	25.165	0.679	80.119	2.507	6.8	8.005
sof-miR171c-5p	166	6	173	22	31	33	14.018	0.51	14.499	1.838	2.738	2.935
sof-miR171d-3p	61	10	68	51	59	166	5.151	0.849	5.699	4.262	5.21	14.764
sof-miR171d-5p	15	0	3	6	15	2	1.267	0	0.251	0.501	1.325	0.178
sof-miR171e-5p	1	0	1	0	0	2	0.084	0	0.084	0	0	0.178
sof-miR171f-3p	36	8	98	108	32	141	3.04	0.679	8.213	9.025	2.826	12.541
sof-miR172-3p	59	5	56	64	31	53	4.982	0.425	4.693	5.348	2.738	4.714
sof-miR172-5p	69	1	29	58	88	76	5.827	0.085	2.43	4.847	7.771	6.759
sof-miR2275-5p	34	20	1	3	71	14	2.871	1.699	0.084	0.251	6.27	1.245
sof-miR319-3p	4791	19556	1955	69	15	17	404.58	1660.9	163.84	5.766	1.325	1.512
sof-miR390-3p	20	3	6	31	20	34	1.689	0.255	0.503	2.59	1.766	3.024
sof-miR390-5p	179	31	258	531	420	757	15.116	2.633	21.622	44.373	37.09	67.328
sof-miR393-3p	110	37	360	71	119	237	9.289	3.142	30.17	5.933	10.509	21.079
sof-miR393-5p	539	141	1795	295	354	938	45.516	11.975	150.43	24.651	31.261	83.426
sof-miR394-5p	57	20	58	58	37	68	4.813	1.699	4.861	4.847	3.267	6.048
sof-miR395a-3p	23	13	67	20	104	141	1.942	1.104	5.615	1.671	9.184	12.541
sof-miR395a-5p	0	0	7	2	18	1	0	0	0.587	0.167	1.59	0.089
sof-miR395b-3p	0	0	0	0	2	4	0	0	0	0	0.177	0.356
sof-miR395b-5p	23	13	65	20	91	141	1.942	1.104	5.447	1.671	8.036	12.541
sof-miR395c-5p	23	11	67	20	104	141	1.942	0.934	5.615	1.671	9.184	12.541
sof-miR396a-3p	21	0	38	6	38	30	1.773	0	3.185	0.501	3.356	2.668
sof-miR396a-5p	7760	359	75303	1304	10499	18033	655.3	30.491	6310.9	108.97	927.15	1603.9
sof-miR396b-3p	11	0	22	1	0	12	0.929	0	1.844	0.084	0	1.067
sof-miR396b-5p	7746	359	75516	1296	10500	18030	654.12	30.491	6328.7	108.3	927.24	1603.6
sof-miR396c-3p	37	0	308	14	55	77	3.124	0	25.812	1.17	4.857	6.848
sof-miR408-3p	5	2	217	10	61	130	0.422	0.17	18.186	0.836	5.387	11.562
sof-miR408-5p	1	0	54	6	9	47	0.084	0	4.526	0.501	0.795	4.18
sof-miR437a-3p	1	1	2	2	2	5	0.084	0.085	0.168	0.167	0.177	0.445
sof-miR437b-3p	61	23	94	61	47	57	5.151	1.953	7.878	5.097	4.151	5.07
sof-miR528-3p	2	0	4	2	7	8	0.169	0	0.335	0.167	0.618	0.712
sof-miR528-5p	0	1	3	12	6	3	0	0.085	0.251	1.003	0.53	0.267
sof-miR5384-3p	18	0	115	10	0	12	1.52	0	9.638	0.836	0	1.067
sof-miR5564a-3p	28	11	10	24	16	20	2.364	0.934	0.838	2.006	1.413	1.779
sof-miR5564a-5p	3483	838	2905	4672	8211	2409	294.13	71.173	243.46	390.41	725.1	214.26
sof-miR5564b-3p	102	46	116	113	32	42	8.613	3.907	9.722	9.443	2.826	3.736

sof-miR5564b-5p	1050	260	595	794	1022	769	88.668	22.082	49.865	66.35	90.252	68.395
sof-miR5564c-5p	3772	868	3014	4915	8628	2597	318.53	73.721	252.59	410.72	761.93	230.98
sof-miR5568a-5p	13	1	15	20	6	10	1.098	0.085	1.257	1.671	0.53	0.889
sof-miR5568b-5p	166	57	275	254	305	252	14.018	4.841	23.047	21.225	26.934	22.413
sof-miR5568c-5p	0	0	1	6	2	6	0	0	0.084	0.501	0.177	0.534
sof-miR5568d-3p	30	5	34	28	35	34	2.533	0.425	2.849	2.34	3.091	3.024
sof-miR5568e-3p	0	0	0	2	0	0	0	0	0	0.167	0	0
sof-miR5568e-5p	114	49	165	187	207	147	9.627	4.162	13.828	15.626	18.28	13.074
sof-miR6223a-3p	1	0	0	1	7	1	0.084	0	0	0.084	0.618	0.089
sof-miR6223b-5p	1	0	0	6	0	0	0.084	0	0	0.501	0	0
sof-miRN001-5p	1	1	0	0	3	0	0.084	0.085	0	0	0.265	0
sof-miRN002-3p	1	0	0	2	1	0	0.084	0	0	0.167	0.088	0
sof-miRN002-5p	4	0	5	12	12	5	0.338	0	0.419	1.003	1.06	0.445
sof-miRN003-3p	0	3	3	5	0	3	0	0.255	0.251	0.418	0	0.267
sof-miRN003-5p	62	1	3	6	2	17	5.236	0.085	0.251	0.501	0.177	1.512
sof-miRN004-3p	12	4	1	14	14	12	1.013	0.34	0.084	1.17	1.236	1.067
sof-miRN005-3p	24	292	608	923	2481	46	2.027	24.8	50.954	77.13	219.09	4.091
sof-miRN005-5p	0	4	6	8	1	0	0	0.34	0.503	0.669	0.088	0
sof-miRN006-3p	2	0	0	0	0	0	0.169	0	0	0	0	0
sof-miRN006-5p	3	1	0	0	0	0	0.253	0.085	0	0	0	0
sof-miRN007-5p	166	57	275	254	305	252	14.018	4.841	23.047	21.225	26.934	22.413
sof-miRN008-3p	0	0	0	0	0	3	0	0	0	0	0	0.267
sof-miRN009-5p	1	1	0	6	2	3	0.084	0.085	0	0.501	0.177	0.267
sof-miRN010-5p	57	28	172	149	92	76	4.813	2.378	14.415	12.451	8.124	6.759
sof-miRN011-3p	2	0	0	5	5	6	0.169	0	0	0.418	0.442	0.534
sof-miRN013-3p	17	17	6	0	20	5	1.436	1.444	0.503	0	1.766	0.445
sof-miRN013-5p	5	23	1	4	1	0	0.422	1.953	0.084	0.334	0.088	0
sof-miRN014-3p	0	5	81	46	76	4	0	0.425	6.788	3.844	6.711	0.356
sof-miRN014-5p	2	131	442	269	477	40	0.169	11.126	37.042	22.479	42.123	3.558
sof-miRN015-3p	0	0	0	0	0	1	0	0	0	0	0	0.089
sof-miRN016-3p	8	2	8	12	38	15	0.676	0.17	0.67	1.003	3.356	1.334
sof-miRN017-3p	27	9	11	13	6	27	2.28	0.764	0.922	1.086	0.53	2.401
sof-miRN018-5p	31	69	53	62	47	26	2.618	5.86	4.442	5.181	4.151	2.312
sof-miRN019-3p	28	22	14	14	20	10	2.364	1.869	1.173	1.17	1.766	0.889
sof-miRN020-3p	20	43	10	18	8	10	1.689	3.652	0.838	1.504	0.706	0.889
sof-miRN021-5p	9	7	8	7	4	13	0.76	0.595	0.67	0.585	0.353	1.156
sof-miRN022-5p	13	2	5	7	9	9	1.098	0.17	0.419	0.585	0.795	0.8
sof-miRN023-5p	42	27	33	18	20	16	3.547	2.293	2.766	1.504	1.766	1.423
sof-miRN024-3p	2	1	3	11	2	2	0.169	0.085	0.251	0.919	0.177	0.178
sof-miRN024-5p	0	0	1	1	2	0	0	0	0.084	0.084	0.177	0
sof-miRN026-3p	24	6	17	26	20	23	2.027	0.51	1.425	2.173	1.766	2.046
sof-miRN027-5p	79	17	133	104	133	131	6.671	1.444	11.146	8.691	11.745	11.651
sof-miRN028-3p	17	4	79	39	30	19	1.436	0.34	6.621	3.259	2.649	1.69
sof-miRN028-5p	9	3	15	11	20	40	0.76	0.255	1.257	0.919	1.766	3.558
sof-miRN029-5p	24	5	47	16	26	19	2.027	0.425	3.939	1.337	2.296	1.69
sof-miRN030-5p	2	3	0	3	1	2	0.169	0.255	0	0.251	0.088	0.178
sof-miRN031-3p	7	8	12	25	32	11	0.591	0.679	1.006	2.089	2.826	0.978
sof-miRN032-5p	4	0	12	0	1	0	0.338	0	1.006	0	0.088	0
sof-miRN033-3p	12	0	3	1	13	16	1.013	0	0.251	0.084	1.148	1.423
sof-miRN034-3p	7	0	0	0	2	2	0.591	0	0	0	0.177	0.178
sof-miRN035-3p	0	0	13	0	0	0	0	0	1.089	0	0	0
sof-miRN036-3p	0	0	0	0	0	2	0	0	0	0	0	0.178
sof-miRN038-5p	1	2	1	5	0	2	0.084	0.17	0.084	0.418	0	0.178
sof-miRN039-5p	3	7	0	2	4	3	0.253	0.595	0	0.167	0.353	0.267
sof-miRN041-3p	0	0	18	1	5	0	0	0	1.509	0.084	0.442	0
sof-miRN042-5p	5	0	2	0	19	1	0.422	0	0.168	0	1.678	0.089
sof-miRN043-5p	0	0	0	1	0	4	0	0	0	0.084	0	0.356
sof-miRN044-5p	22	15	43	7	20	1	1.858	1.274	3.604	0.585	1.766	0.089
sof-miRN045-3p	16	15	24	10	4	21	1.351	1.274	2.011	0.836	0.353	1.868
sof-miRN047-3p	0	0	1	0	0	1	0	0	0.084	0	0	0.089
sof-miRN048-3p	10	48	48	34	41	67	0.844	4.077	4.023	2.841	3.621	5.959
sof-miRN049-3p	14	1	0	0	0	0	1.182	0.085	0	0	0	0
sof-miRN049-5p	3	0	0	0	0	0	0.253	0	0	0	0	0
sof-miRN050-5p	20	24	41	13	14	26	1.689	2.038	3.436	1.086	1.236	2.312
sof-miRN051-3p	0	0	0	12	6	0	0	0	0	1.003	0.53	0
sof-miRN054-3p	225	165	1383	96	221	717	19	14.014	115.9	8.022	19.516	63.771
sof-miRN055-3p	0	1	9	5	3	6	0	0.085	0.754	0.418	0.265	0.534
sof-miRN056-3p	23	18	68	58	97	58	1.942	1.529	5.699	4.847	8.566	5.159
sof-miRN057-5p	50	21	208	59	39	38	4.222	1.784	17.432	4.93	3.444	3.38
sof-miRN058-5p	0	1	7	0	0	0	0	0.085	0.587	0	0	0
sof-miRN059-3p	1	0	0	0	0	1	0.084	0	0	0	0	0.089
sof-miRN060-3p	100	114	66	106	52	46	8.445	9.682	5.531	8.858	4.592	4.091
sof-miRN061-5p	8	7	33	22	16	10	0.676	0.595	2.766	1.838	1.413	0.889
sof-miRN062-3p	18	21	23	26	17	30	1.52	1.784	1.928	2.173	1.501	2.668
sof-miRN063-3p	1	6	13	3	5	10	0.084	0.51	1.089	0.251	0.442	0.889
sof-miRN064-3p	0	0	0	0	0	1	0	0	0	0	0	0.089
sof-miRN065-3p	0	3	2	2	20	14	0	0.255	0.168	0.167	1.766	1.245
sof-miRN066-3p	7	13	12	10	24	18	0.591	1.104	1.006	0.836	2.119	1.601
sof-miRN070-3p	12	17	2	3	4	4	1.013	1.444	0.168	0.251	0.353	0.356
sof-miRN071-5p	0	0	0	1	0	1	0	0	0	0.084	0	0.089
sof-miRN072-3p	4	2	7	16	1	1	0.338	0.17	0.587	1.337	0.088	0.089
sof-miRN073-5p	11	0	0	0	1	0	0.929	0	0	0	0.088	0
sof-miRN074-3p	11	1	18	18	12	1	0.929	0.085	1.509	1.504	1.06	0.089
sof-miRN075-3p	0	0	1	4	2	11	0	0	0.084	0.334	0.177	0.978
sof-miRN078-3p	8	1	0	4	0	3	0.676	0.085	0	0.334	0	0.267
sof-miRN078-5p	17	7	4	22	12	34	1.436	0.595	0.335	1.838	1.06	3.024
sof-miRN079-3p	8	4	8	15	5	0	0.676	0.34	0.67	1.253	0.442	0

sof-miRN080-3p	0	0	0	0	7	0	0	0	0	0	0.618	0
sof-miRN081-3p	3	7	12	8	10	4	0.253	0.595	1.006	0.669	0.883	0.356
sof-miRN083-3p	1	1	17	6	3	12	0.084	0.085	1.425	0.501	0.265	1.067
sof-miRN084-5p	0	11	0	17	0	0	0	0.934	0	1.421	0	0
sof-miRN085-5p	12	21	1	17	6	6	1.013	1.784	0.084	1.421	0.53	0.534
sof-miRN086-3p	3	2	13	8	0	3	0.253	0.17	1.089	0.669	0	0.267
sof-miRN088-3p	19	2	6	5	24	16	1.604	0.17	0.503	0.418	2.119	1.423
sof-miRN089-5p	36	7	3	3	14	66	3.04	0.595	0.251	0.251	1.236	5.87
sof-miRN090-3p	0	4	14	0	1	0	0	0.34	1.173	0	0.088	0
sof-miRN092-3p	26	31	35	18	31	20	2.196	2.633	2.933	1.504	2.738	1.779
sof-miRN093-3p	24	4	22	9	43	37	2.027	0.34	1.844	0.752	3.797	3.291
sof-miRN094-3p	1	0	1	0	4	0	0.084	0	0.084	0	0.353	0
sof-miRN095-5p	2	4	6	5	3	3	0.169	0.34	0.503	0.418	0.265	0.267
sof-miRN096-5p	66	61	68	59	74	54	5.573	5.181	5.699	4.93	6.535	4.803
sof-miRN097-5p	534	281	294	277	90	113	45.094	23.866	24.639	23.147	7.948	10.05
sof-miRN098-3p	1	1	0	0	0	0	0.084	0.085	0	0	0	0
sof-miRN099-5p	158	85	287	190	212	127	13.342	7.219	24.052	15.877	18.721	11.295
sof-miRN100-3p	5	8	3	0	5	5	0.422	0.679	0.251	0	0.442	0.445
sof-miRN101-3p	22	29	8	3	8	11	1.858	2.463	0.67	0.251	0.706	0.978
sof-miRN102-5p	0	0	0	1	0	0	0	0	0	0.084	0	0
sof-miRN107-3p	31	27	21	14	13	18	2.618	2.293	1.76	1.17	1.148	1.601
sof-miRN108-3p	27	19	70	18	12	4	2.28	1.614	5.866	1.504	1.06	0.356
sof-miRN109-5p	34	4	16	60	47	18	2.871	0.34	1.341	5.014	4.151	1.601
sof-miRN110-3p	30	19	11	9	24	13	2.533	1.614	0.922	0.752	2.119	1.156
sof-miRN111-3p	2	1	2	1	0	0	0.169	0.085	0.168	0.084	0	0
sof-miRN112-3p	50	26	99	30	28	43	4.222	2.208	8.297	2.507	2.473	3.824
sof-miRN113-3p	13	7	12	6	10	2	1.098	0.595	1.006	0.501	0.883	0.178
sof-miRN115-5p	116	76	204	82	114	83	9.796	6.455	17.097	6.852	10.067	7.382
sof-miRN116-5p	3	0	0	0	0	0	0.253	0	0	0	0	0
sof-miRN117-5p	1	4	8	0	0	1	0.084	0.34	0.67	0	0	0.089
sof-miRN118-5p	14	7	14	22	3	14	1.182	0.595	1.173	1.838	0.265	1.245
sof-miRN119-3p	0	0	4	8	4	4	0	0	0.335	0.669	0.353	0.356
sof-miRN119-5p	1	1	10	3	1	5	0.084	0.085	0.838	0.251	0.088	0.445
sof-miRN120-3p	16	15	44	0	34	0	1.351	1.274	3.687	0	3.002	0
sof-miRN120-5p	8	29	1	1	4	2	0.676	2.463	0.084	0.084	0.353	0.178
sof-miRN123-5p	22	7	13	10	11	2	1.858	0.595	1.089	0.836	0.971	0.178
sof-miRN124-3p	1	0	0	0	0	0	0.084	0	0	0	0	0
sof-miRN125-5p	2	6	0	3	4	0	0.169	0.51	0	0.251	0.353	0
sof-miRN128-3p	0	0	1	0	1	0	0	0	0.084	0	0.088	0
sof-miRN129-5p	27	0	36	13	8	19	2.28	0	3.017	1.086	0.706	1.69
sof-miRN130-3p	4	0	2	1	0	10	0.338	0	0.168	0.084	0	0.889
sof-miRN130-5p	0	0	2	0	0	1	0	0	0.168	0	0	0.089
sof-miRN131-5p	11	6	3	3	1	1	0.929	0.51	0.251	0.251	0.088	0.089
sof-miRN133-5p	0	0	0	1	0	0	0	0	0	0.084	0	0
sof-miRN134-3p	71	152	146	49	93	506	5.996	12.91	12.236	4.095	8.213	45.004
sof-miRN134-5p	2	12	24	12	5	59	0.169	1.019	2.011	1.003	0.442	5.248
sof-miRN136-3p	5	6	10	4	1	3	0.422	0.51	0.838	0.334	0.088	0.267
sof-miRN137-3p	5	6	10	4	1	3	0.422	0.51	0.838	0.334	0.088	0.267
sof-miRN138-5p	0	1	70	104	0	0	0	0.085	5.866	8.691	0	0
sof-miRN139-3p	30	0	59	13	8	20	2.533	0	4.945	1.086	0.706	1.779
sof-miRN140-3p	44	2	0	0	2	14	3.716	0.17	0	0	0.177	1.245
sof-miRN141-3p	394	18	0	222	0	207	33.272	1.529	0	18.551	0	18.411
sof-miRN142-5p	12	1	16	15	1	11	1.013	0.085	1.341	1.253	0.088	0.978
sof-miRN143-5p	12	1	16	15	1	11	1.013	0.085	1.341	1.253	0.088	0.978
sof-miRN144-3p	30	0	59	13	8	20	2.533	0	4.945	1.086	0.706	1.779
sof-miRN144-5p	9	0	22	12	0	5	0.76	0	1.844	1.003	0	0.445
sof-miRN145-3p	16	7	1	1	107	1	1.351	0.595	0.084	0.084	9.449	0.089
sof-miRN146-5p	0	2	0	16	0	0	0	0.17	0	1.337	0	0
sof-miRN147-5p	2	2	0	1	0	1	0.169	0.17	0	0.084	0	0.089
sof-miRN148-5p	0	0	0	0	1	1	0	0	0	0	0.088	0.089
sof-miRN149-3p	3	2	12	11	6	2	0.253	0.17	1.006	0.919	0.53	0.178
sof-miRN150-3p	40	8	20	22	38	18	3.378	0.679	1.676	1.838	3.356	1.601
sof-miRN151-3p	1	0	1	2	0	0	0.084	0	0.084	0.167	0	0
sof-miRN154-5p	114	126	293	142	199	97	9.627	10.701	24.555	11.866	17.573	8.627
sof-miRN155-5p	114	126	293	142	199	97	9.627	10.701	24.555	11.866	17.573	8.627
sof-miRN157-5p	1	1	12	9	4	3	0.084	0.085	1.006	0.752	0.353	0.267
sof-miRN158-3p	0	0	0	1	0	0	0	0	0	0.084	0	0
sof-miRN159-3p	0	3	0	0	13	0	0	0.255	0	0	1.148	0
sof-miRN160-3p	10	7	21	9	2	3	0.844	0.595	1.76	0.752	0.177	0.267
sof-miRN162-3p	10	0	3	1	0	0	0.844	0	0.251	0.084	0	0
sof-miRN162-5p	1	0	0	0	0	1	0.084	0	0	0	0	0.089
sof-miRN164-3p	0	1	0	0	2	4	0	0.085	0	0	0.177	0.356
sof-miRN164-5p	168	84	0	98	64	111	14.187	7.134	0	8.189	5.652	9.872
sof-miRN165-3p	2	3	0	2	12	3	0.169	0.255	0	0.167	1.06	0.267
sof-miRN167-3p	0	0	171	0	2	0	0	0	14.331	0	0.177	0
sof-miRN168-5p	0	0	0	3	0	0	0	0	0	0.251	0	0
sof-miRN174-3p	1	0	16	21	0	3	0.084	0	1.341	1.755	0	0.267
sof-miRN175-5p	0	1	0	0	0	0	0	0.085	0	0	0	0
sof-miRN177-3p	15	4	32	18	7	34	1.267	0.34	2.682	1.504	0.618	3.024
sof-miRN183-3p	4	1	0	2	0	0	0.338	0.085	0	0.167	0	0
sof-miRN183-5p	2	12	2	8	0	2	0.169	1.019	0.168	0.669	0	0.178
sof-miRN184-3p	97	88	417	104	94	168	8.191	7.474	34.947	8.691	8.301	14.942
sof-miRN185-5p	1	0	1	0	1	1	0.084	0	0.084	0	0.088	0.089
sof-miRN187-3p	2	0	0	1	0	0	0.169	0	0	0.084	0	0
sof-miRN188-5p	0	0	0	1	0	0	0	0	0	0.084	0	0
sof-miRN189-5p	9	7	21	2	11	7	0.76	0.595	1.76	0.167	0.971	0.623
sof-miRN190-5p	34	9	1	7	19	3	2.871	0.764	0.084	0.585	1.678	0.267

sof-miRN191-5p	49	6	62	61	125	33	4.138	0.51	5.196	5.097	11.039	2.935
sof-miRN192-3p	8	3	7	5	24	21	0.676	0.255	0.587	0.418	2.119	1.868
sof-miRN193-3p	67	32	320	165	167	114	5.658	2.718	26.818	13.788	14.748	10.139
sof-miRN193-5p	61	13	150	127	245	78	5.151	1.104	12.571	10.613	21.636	6.937
sof-miRN194-3p	17	24	14	23	18	20	1.436	2.038	1.173	1.922	1.59	1.779
sof-miRN196-5p	21	13	16	7	9	7	1.773	1.104	1.341	0.585	0.795	0.623
sof-miRN197-5p	11	7	47	1	37	1	0.929	0.595	3.939	0.084	3.267	0.089
sof-miRN198-5p	17	0	16	17	10	11	1.436	0	1.341	1.421	0.883	0.978
sof-miRN199-3p	13	5	6	6	3	8	1.098	0.425	0.503	0.501	0.265	0.712
sof-miRN199-5p	1	0	1	0	0	0	0.084	0	0.084	0	0	0
sof-miRN201-3p	231	322	507	877	308	325	19.507	27.348	42.49	73.286	27.199	28.906
sof-miRN202-3p	39	15	106	21	24	6	3.293	1.274	8.883	1.755	2.119	0.534
sof-miRN203-5p	2	0	1	2	1	0	0.169	0	0.084	0.167	0.088	0
sof-miRN204-5p	2	0	0	2	30	0	0.169	0	0	0.167	2.649	0
sof-miRN205-5p	4	0	0	1	2	6	0.338	0	0	0.084	0.177	0.534
sof-miRN207-5p	84	186	63	191	39	35	7.093	15.797	5.28	15.961	3.444	3.113
sof-miRN210-5p	5	4	4	7	1	2	0.422	0.34	0.335	0.585	0.088	0.178
sof-miRN212-3p	0	1	0	2	0	0	0	0.085	0	0.167	0	0
sof-miRN215-5p	17	30	0	0	20	0	1.436	2.548	0	0	1.766	0
sof-miRN218-3p	15	4	7	16	13	8	1.267	0.34	0.587	1.337	1.148	0.712
sof-miRN220-5p	9	25	4	6	1	6	0.76	2.123	0.335	0.501	0.088	0.534
sof-miRN222-5p	31	19	93	57	38	65	2.618	1.614	7.794	4.763	3.356	5.781
sof-miRN223-5p	20	24	41	13	14	26	1.689	2.038	3.436	1.086	1.236	2.312
sof-miRN225-5p	1	1	0	2	0	0	0.084	0.085	0	0.167	0	0
sof-miRN226-5p	5	3	2	14	12	3	0.422	0.255	0.168	1.17	1.06	0.267
sof-miRN228-3p	9	3	16	4	6	4	0.76	0.255	1.341	0.334	0.53	0.356
sof-miRN229-5p	2	0	12	4	2	0	0.169	0	1.006	0.334	0.177	0
sof-miRN230-5p	2	0	12	4	2	0	0.169	0	1.006	0.334	0.177	0
sof-miRN232-3p	2	5	10	6	3	5	0.169	0.425	0.838	0.501	0.265	0.445
sof-miRN233-5p	50	4	3	9	2	17	4.222	0.34	0.251	0.752	0.177	1.512
sof-miRN234-5p	0	0	1	0	0	0	0	0	0.084	0	0	0
sof-miRN235-5p	61	60	168	32	83	77	5.151	5.096	14.079	2.674	7.33	6.848
sof-miRN236-5p	85	74	131	100	104	49	7.178	6.285	10.979	8.356	9.184	4.358
sof-miRN237-3p	117	75	186	68	108	62	9.88	6.37	15.588	5.682	9.537	5.514
sof-miRN238-3p	12	11	29	21	10	15	1.013	0.934	2.43	1.755	0.883	1.334
sof-miRN239-3p	13	0	1	3	3	5	1.098	0	0.084	0.251	0.265	0.445
sof-miRN240-5p	1	0	0	0	0	2	0.084	0	0	0	0	0.178
sof-miRN241-3p	4	5	0	1	0	3	0.338	0.425	0	0.084	0	0.267
sof-miRN243-3p	988	0	1	0	360	265	83.433	0	0.084	0	31.791	23.569
sof-miRN245-5p	1	84	6	13	10	15	0.084	7.134	0.503	1.086	0.883	1.334
sof-miRN246-3p	14	9	5	13	3	3	1.182	0.764	0.419	1.086	0.265	0.267
sof-miRN249-5p	22	8	14	12	4	7	1.858	0.679	1.173	1.003	0.353	0.623
sof-miRN250-3p	3	7	0	2	5	3	0.253	0.595	0	0.167	0.442	0.267
sof-miRN251-3p	1	3	0	0	0	2	0.084	0.255	0	0	0	0.178
sof-miRN252-3p	3	4	8	2	11	1	0.253	0.34	0.67	0.167	0.971	0.089
sof-miRN254-3p	1	1	0	0	19	0	0.084	0.085	0	0	1.678	0
sof-miRN254-5p	110	60	0	0	182	3	9.289	5.096	0	0	16.072	0.267
sof-miRN255-3p	20	0	4	15	19	20	1.689	0	0.335	1.253	1.678	1.779
sof-miRN256-3p	20	0	4	15	23	24	1.689	0	0.335	1.253	2.031	2.135
sof-miRN259-3p	14	1	7	7	10	12	1.182	0.085	0.587	0.585	0.883	1.067
sof-miRN259-5p	162	26	313	127	229	160	13.68	2.208	26.231	10.613	20.223	14.231
sof-miRN260-3p	14	1	7	7	10	12	1.182	0.085	0.587	0.585	0.883	1.067
sof-miRN260-5p	162	26	313	127	229	160	13.68	2.208	26.231	10.613	20.223	14.231
sof-miRN261-5p	2	23	22	18	22	16	0.169	1.953	1.844	1.504	1.943	1.423
sof-miRN262-5p	2	23	22	18	22	16	0.169	1.953	1.844	1.504	1.943	1.423
sof-miRN264-5p	0	0	0	2	0	0	0	0	0	0.167	0	0
sof-miRN265-5p	1	0	19	3	3	2	0.084	0	1.592	0.251	0.265	0.178
sof-miRN266-3p	1	0	0	0	0	0	0.084	0	0	0	0	0
sof-miRN267-3p	5	0	18	1	0	14	0.422	0	1.509	0.084	0	1.245
sof-miRN267-5p	28	0	94	9	29	38	2.364	0	7.878	0.752	2.561	3.38
sof-miRN268-3p	0	0	1	0	0	0	0	0	0.084	0	0	0
sof-miRN269-5p	8	8	5	6	5	13	0.676	0.679	0.419	0.501	0.442	1.156
sof-miRN271-3p	26	20	8	8	12	8	2.196	1.699	0.67	0.669	1.06	0.712
sof-miRN272-5p	8	8	14	18	1	15	0.676	0.679	1.173	1.504	0.088	1.334
sof-miRN273-3p	44	2	0	0	2	14	3.716	0.17	0	0	0.177	1.245
sof-miRN275-3p	4	2	7	16	1	1	0.338	0.17	0.587	1.337	0.088	0.089
sof-miRN276-3p	33	6	53	19	46	27	2.787	0.51	4.442	1.588	4.062	2.401
sof-miRN276-5p	7	2	14	2	3	9	0.591	0.17	1.173	0.167	0.265	0.8
sof-miRN279-3p	0	1	4	2	0	0	0	0.085	0.335	0.167	0	0
sof-miRN282-5p	3	0	0	0	0	0	0.253	0	0	0	0	0
sof-miRN283-3p	2	19	1	2	0	4	0.169	1.614	0.084	0.167	0	0.356
sof-miRN284-3p	19	35	37	14	15	10	1.604	2.973	3.101	1.17	1.325	0.889
sof-miRN285-3p	65	41	166	102	136	97	5.489	3.482	13.912	8.524	12.01	8.627
sof-miRN286-3p	50	4	44	38	67	24	4.222	0.34	3.687	3.175	5.917	2.135
sof-miRN287-3p	21	0	0	3	0	16	1.773	0	0	0.251	0	1.423
sof-miRN288-5p	20	28	29	32	21	28	1.689	2.378	2.43	2.674	1.854	2.49
sof-miRN289-5p	1	2	13	0	15	0	0.084	0.17	1.089	0	1.325	0
sof-miRN290-5p	1	0	59	2	0	0	0.084	0	4.945	0.167	0	0
sof-miRN292-3p	0	0	16	4	0	0	0	0	1.341	0.334	0	0
sof-miRN293-3p	5	2	12	10	2	13	0.422	0.17	1.006	0.836	0.177	1.156
sof-miRN294-3p	8	7	26	15	8	9	0.676	0.595	2.179	1.253	0.706	0.8
sof-miRN296-3p	5	2	3	3	5	5	0.422	0.17	0.251	0.251	0.442	0.445
sof-miRN296-5p	5	1	1	8	6	2	0.422	0.085	0.084	0.669	0.53	0.178
sof-miRN297-5p	20	3	15	16	16	6	1.689	0.255	1.257	1.337	1.413	0.534
sof-miRN298-5p	0	0	0	1	3	2	0	0	0	0.084	0.265	0.178
sof-miRN299-5p	12	10	13	24	13	17	1.013	0.849	1.089	2.006	1.148	1.512
sof-miRN300-5p	23	12	30	28	15	22	1.942	1.019	2.514	2.34	1.325	1.957

sof-miRN301-5p	0	0	2	5	1	12	0	0	0.168	0.418	0.088	1.067
sof-miRN302-3p	1	1	0	1	0	2	0.084	0.085	0	0.084	0	0.178
sof-miRN303-5p	12	141	7	1	27	17	1.013	11.975	0.587	0.084	2.384	1.512
sof-miRN304-3p	0	0	1	0	1	1	0	0	0.084	0	0.088	0.089
sof-miRN305-5p	32	48	13	25	11	14	2.702	4.077	1.089	2.089	0.971	1.245
sof-miRN306-3p	27	0	36	13	8	19	2.28	0	3.017	1.086	0.706	1.69
sof-miRN307-3p	4	1	3	1	2	1	0.338	0.085	0.251	0.084	0.177	0.089
sof-miRN308-3p	8	8	26	11	6	11	0.676	0.679	2.179	0.919	0.53	0.978
sof-miRN310-3p	17	24	3	10	9	9	1.436	2.038	0.251	0.836	0.795	0.8
sof-miRN312-3p	7	1	11	6	14	2	0.591	0.085	0.922	0.501	1.236	0.178
sof-miRN313-5p	0	0	0	0	1	1	0	0	0	0	0.088	0.089
sof-miRN314-5p	0	4	9	9	4	3	0	0.34	0.754	0.752	0.353	0.267
sof-miRN316-3p	0	0	2	0	12	0	0	0	0.168	0	1.06	0
sof-miRN318-3p	1	0	2	3	3	2	0.084	0	0.168	0.251	0.265	0.178
sof-miRN319-5p	1	11	0	35	39	0	0.084	0.934	0	2.925	3.444	0
sof-miRN320-3p	0	1	0	0	0	0	0	0.085	0	0	0	0
sof-miRN320-5p	4	2	1	0	4	1	0.338	0.17	0.084	0	0.353	0.089
sof-miRN321-3p	1	0	0	1	0	2	0.084	0	0	0.084	0	0.178
sof-miRN322-5p	6	4	8	2	3	2	0.507	0.34	0.67	0.167	0.265	0.178
sof-miRN323-3p	3	13	3	14	4	3	0.253	1.104	0.251	1.17	0.353	0.267
sof-miRN324-5p	11	7	30	16	22	10	0.929	0.595	2.514	1.337	1.943	0.889
sof-miRN325-5p	117	65	106	101	169	41	9.88	5.521	8.883	8.44	14.924	3.647
sof-miRN326-3p	0	0	0	1	0	0	0	0	0	0.084	0	0
sof-miRN327-5p	4	16	67	2	23	0	0.338	1.359	5.615	0.167	2.031	0
sof-miRN328-3p	1	3	5	0	0	1	0.084	0.255	0.419	0	0	0.089
sof-miRN329-3p	1	3	5	0	0	1	0.084	0.255	0.419	0	0	0.089
sof-miRN330-3p	6	5	32	21	5	6	0.507	0.425	2.682	1.755	0.442	0.534
sof-miRN331-3p	58	5	44	89	53	27	4.898	0.425	3.687	7.437	4.68	2.401
sof-miRN331-5p	0	0	0	0	1	1	0	0	0	0	0.088	0.089
sof-miRN332-5p	0	0	15	0	0	0	0	0	1.257	0	0	0
sof-miRN333-3p	5	13	3	6	0	4	0.422	1.104	0.251	0.501	0	0.356
sof-miRN334-3p	7	3	5	14	1	6	0.591	0.255	0.419	1.17	0.088	0.534
sof-miRN335-5p	17	7	4	22	12	34	1.436	0.595	0.335	1.838	1.06	3.024
sof-miRN337-3p	4	1	18	3	7	8	0.338	0.085	1.509	0.251	0.618	0.712
sof-miRN338-3p	4	6	2	9	10	16	0.338	0.51	0.168	0.752	0.883	1.423
sof-miRN339-5p	45	57	164	154	75	62	3.8	4.841	13.744	12.869	6.623	5.514
sof-miRN340-5p	0	0	21	0	0	0	0	0	1.76	0	0	0
sof-miRN343-3p	3776	1418	1775	13864	6789	4940	318.87	120.43	148.76	1158.5	599.53	439.37
sof-miRN343-5p	9374	3233	9886	8683	10759	13511	791.6	274.59	828.51	725.59	950.11	1201.7
sof-miRN344-5p	7	10	12	1	9	12	0.591	0.849	1.006	0.084	0.795	1.067
sof-miRN345-5p	26	18	39	15	11	18	2.196	1.529	3.268	1.253	0.971	1.601
sof-miRN348-3p	0	0	0	1	0	1	0	0	0	0.084	0	0.089
sof-miRN349-3p	10	1	1	6	4	0	0.844	0.085	0.084	0.501	0.353	0
sof-miRN350-5p	1	4	3	10	12	8	0.084	0.34	0.251	0.836	1.06	0.712
sof-miRN351-3p	2	1	3	4	4	0	0.169	0.085	0.251	0.334	0.353	0
sof-miRN351-5p	1	0	1	0	6	0	0.084	0	0.084	0	0.53	0
sof-miRN352-5p	0	0	0	1	1	0	0	0	0	0.084	0.088	0
sof-miRN353-3p	2	0	0	0	1	1	0.169	0	0	0	0.088	0.089
sof-miRN353-5p	1	0	0	0	0	0	0.084	0	0	0	0	0
sof-miRN354-5p	1	0	0	0	0	0	0.084	0	0	0	0	0
sof-miRN355-5p	0	0	1	2	0	0	0	0	0.084	0.167	0	0
sof-miRN356-5p	8	0	0	12	0	3	0.676	0	0	1.003	0	0.267
sof-miRN357-5p	0	0	0	0	1	0	0	0	0	0	0.088	0
sof-miRN358-3p	0	0	0	1	0	0	0	0	0	0.084	0	0
sof-miRN358-5p	9	2	18	15	3	8	0.76	0.17	1.509	1.253	0.265	0.712
sof-miRN359-5p	10	7	5	5	9	3	0.844	0.595	0.419	0.418	0.795	0.267
sof-miRN360-5p	36	9	11	3	2	7	3.04	0.764	0.922	0.251	0.177	0.623
sof-miRN361-3p	72	67	314	90	134	22	6.08	5.69	26.315	7.521	11.833	1.957
sof-miRN366-3p	80	105	123	53	60	61	6.756	8.918	10.308	4.429	5.299	5.425
sof-miRN367-3p	14	0	2	20	6	0	1.182	0	0.168	1.671	0.53	0
sof-miRN368-3p	22	11	9	20	8	15	1.858	0.934	0.754	1.671	0.706	1.334
sof-miRN368-5p	2	0	0	0	0	0	0.169	0	0	0	0	0
sof-miRN369-3p	15	12	5	24	24	5	1.267	1.019	0.419	2.006	2.119	0.445
sof-miRN371-3p	2	2	10	3	1	0	0.169	0.17	0.838	0.251	0.088	0
sof-miRN371-5p	1	0	3	0	5	6	0.084	0	0.251	0	0.442	0.534
sof-miRN372-3p	37	43	99	44	26	26	3.124	3.652	8.297	3.677	2.296	2.312
sof-miRN373-5p	37	5	54	19	8	25	3.124	0.425	4.526	1.588	0.706	2.224
sof-miRN374-5p	1	0	1	0	1	1	0.084	0	0.084	0	0.088	0.089
sof-miRN375-3p	37	0	308	14	55	77	3.124	0	25.812	1.17	4.857	6.848
sof-miRN376-5p	0	1	0	2	2	0	0	0.085	0	0.167	0.177	0
sof-miRN377-5p	12	121	16	10	27	20	1.013	10.277	1.341	0.836	2.384	1.779
sof-miRN378-3p	2	3	12	0	1	4	0.169	0.255	1.006	0	0.088	0.356
sof-miRN379-5p	837	229	1433	669	577	806	70.681	19.45	120.1	55.904	50.954	71.686
sof-miRN381-3p	37	57	71	61	56	36	3.124	4.841	5.95	5.097	4.945	3.202
sof-miRN382-5p	3	0	0	0	2	1	0.253	0	0	0	0.177	0.089
sof-miRN383-3p	41	32	11	19	20	3	3.462	2.718	0.922	1.588	1.766	0.267
sof-miRN385-3p	12	26	47	44	52	36	1.013	2.208	3.939	3.677	4.592	3.202
sof-miRN386-5p	27	42	16	12	12	16	2.28	3.567	1.341	1.003	1.06	1.423
sof-miRN389-5p	0	2	0	0	0	0	0	0.17	0	0	0	0
sof-miRN390-3p	3	1	2	1	0	1	0.253	0.085	0.168	0.084	0	0.089
sof-miRN391-3p	3	5	4	3	1	3	0.253	0.425	0.335	0.251	0.088	0.267
sof-miRN392-3p	3	15	4	0	28	13	0.253	1.274	0.335	0	2.473	1.156
sof-miRN393-3p	5	5	23	2	0	5	0.422	0.425	1.928	0.167	0	0.445
sof-miRN394-3p	2	1	14	3	14	3	0.169	0.085	1.173	0.251	1.236	0.267
sof-miRN395-3p	4	0	0	1	1	3	0.338	0	0	0.084	0.088	0.267
sof-miRN396-3p	1305	1197	1318	1565	1908	1092	110.2	101.66	110.46	130.78	168.49	97.123
sof-miRN397-5p	40	24	20	167	82	106	3.378	2.038	1.676	13.955	7.241	9.428

sof-miRN398-3p	29	20	29	22	28	10	2.449	1.699	2.43	1.838	2.473	0.889
sof-miRN401-3p	0	0	0	0	3	0	0	0	0	0	0.265	0
sof-miRN402-3p	4	12	9	24	22	14	0.338	1.019	0.754	2.006	1.943	1.245
sof-miRN403-3p	0	0	0	2	0	0	0	0	0	0.167	0	0
sof-miRN405-5p	19	49	17	39	43	18	1.604	4.162	1.425	3.259	3.797	1.601
sof-miRN406-5p	1	2	0	13	4	4	0.084	0.17	0	1.086	0.353	0.356
sof-miRN407-3p	0	0	0	2	0	0	0	0	0	0.167	0	0
sof-miRN408-3p	1	1	0	0	19	0	0.084	0.085	0	0	1.678	0
sof-miRN408-5p	110	60	0	0	182	3	9.289	5.096	0	0	16.072	0.267

Table S2. KEGG pathway enrichment analysis for differentially expressed miRNAs.

Group	Pathway	ID	Gene_number	p-value	q-value	miRNA
Glycan biosynthesis and metabolism	Glycosylphosphatidylinositol(GPI)-anchor biosynthesis	ko00563	9	7.83E-06	1.20E-03	sof-miR393-3p,sof-miR393-5p
Cell growth and death	Meiosis - yeast	ko04113	9	2.09E-05	3.20E-03	sof-miRN245-5p,sof-miR5384-3p
Signal transduction	Plant hormone signal transduction	ko04075	22	0.000218	3.34E-02	sof-miR171c-3p,sof-miR393-5p,sof-miR5384-3p,sof-miR167a-3p,sof-miRN141-3p
Cell growth and death	Oocyte meiosis	ko04114	9	0.0003	4.59E-02	sof-miR5384-3p,sof-miRN245-5p
Endocrine system	Adipocytokine signaling pathway	ko04920	4	0.002303	3.52E-01	sof-miRN141-3p,sof-miR166d-5p
Transport and catabolism	Regulation of autophagy	ko04140	4	0.002445	3.74E-01	sof-miRN141-3p,sof-miRN245-5p
Cell growth and death	Cell cycle - yeast	ko04111	7	0.002478	3.79E-01	sof-miRN245-5p
Endocrine system	Oxytocin signaling pathway	ko04921	6	0.004325	6.62E-01	sof-miRN141-3p,sof-miR166d-5p,sof-miR5384-3p
Endocrine system	Regulation of lipolysis in adipocytes	ko04923	2	0.007803	1.00E+00	sof-miR5384-3p
Endocrine system	Ovarian steroidogenesis	ko04913	2	0.007803	1.00E+00	sof-miR5384-3p
Sensory system	Taste transduction	ko04742	2	0.009448	1.00E+00	sof-miR5384-3p
Lipid metabolism	Fatty acid elongation	ko00062	3	0.01792	1.00E+00	sof-miR408-3p
Amino acid metabolism	Tryptophan metabolism	ko00380	4	0.01795	1.00E+00	sof-miRN164-5p,sof-miR167a-3p
Glycan biosynthesis and metabolism	Glycosphingolipid biosynthesis - globo series	ko00603	2	0.02642	1.00E+00	sof-miR5384-3p
Biosynthesis of other secondary metabolites	Betalain biosynthesis	ko00965	1	0.02717	1.00E+00	sof-miR408-3p
Excretory system	Vasopressin-regulated water reabsorption	ko04962	2	0.03796	1.00E+00	sof-miR5384-3p
Lipid metabolism	Biosynthesis of unsaturated fatty acids	ko01040	3	0.03808	1.00E+00	sof-miR408-3p
Excretory system	Endocrine and other factor-regulated calcium reabsorption	ko04961	2	0.04217	1.00E+00	sof-miR5384-3p
Nervous system	Cholinergic synapse	ko04725	2	0.04217	1.00E+00	sof-miR5384-3p
Nervous system	Serotonergic synapse	ko04726	2	0.04217	1.00E+00	sof-miR5384-3p
Signal transduction	Hedgehog signaling pathway	ko04340	2	0.04992	1.00E+00	sof-miR5384-3p
Infectious diseases: Parasitic	Amoebiasis	ko05146	2	0.05339	1.00E+00	sof-miR5384-3p
Signal transduction	AMPK signaling pathway	ko04152	4	0.06458	1.00E+00	sof-miR166d-5p,sof-miRN141-3p
Biosynthesis of other secondary metabolites	Tropane, piperidine and pyridine alkaloid biosynthesis	ko00960	2	0.07062	1.00E+00	sof-miRN167-3p
Carbohydrate metabolism	Galactose metabolism	ko00052	4	0.07405	1.00E+00	sof-miR319-3p
Environmental adaptation	Circadian rhythm	ko04710	2	0.08525	1.00E+00	sof-miRN141-3p
Cell growth and death	Cell cycle	ko04110	7	0.09359	1.00E+00	sof-miRN245-5p
Sensory system	Olfactory transduction	ko04740	2	0.1007	1.00E+00	sof-miR5384-3p
Nervous system	Retrograde endocannabinoid signaling	ko04723	2	0.1021	1.00E+00	sof-miR5384-3p
Infectious diseases: Parasitic	African trypanosomiasis	ko05143	1	0.1044	1.00E+00	sof-miR167a-3p
Cellular community	Gap junction	ko04540	2	0.1079	1.00E+00	sof-miR5384-3p
Endocrine system	Progesterone-mediated oocyte maturation	ko04914	3	0.1087	1.00E+00	sof-miR5384-3p,sof-miRN014-3p
Endocrine system	Aldosterone synthesis and secretion	ko04925	2	0.1094	1.00E+00	sof-miR5384-3p
Sensory system	Inflammatory mediator regulation of TRP channels	ko04750	2	0.1168	1.00E+00	sof-miR5384-3p
Environmental adaptation	Circadian entrainment	ko04713	2	0.1414	1.00E+00	sof-miR5384-3p
Metabolism of terpenoids and polyketides	Zeatin biosynthesis	ko00908	3	0.1516	1.00E+00	sof-miR395b-5p,sof-miR395c-5p,sof-miR395a-3p
Global and overview maps	Fatty acid metabolism	ko01212	3	0.1527	1.00E+00	sof-miR408-3p
Environmental adaptation	Plant-pathogen interaction	ko04626	20	0.1642	1.00E+00	sof-miR166e-5p,sof-miR167b-5p,sof-miR393-3p,sof-miR159-5p,sof-miRN014-3p,sof-miRN141-3p,sof-miR396b-5p,sof-miR396a-5p,sof-miR167c-5p,sof-miR167a-5p,sof-miRN245-5p
Endocrine system	Estrogen signaling pathway	ko04915	3	0.1692	1.00E+00	sof-miRN014-3p,sof-miR5384-3p
Nervous system	GABAergic synapse	ko04727	2	0.1717	1.00E+00	sof-miR5384-3p
Replication and repair	Homologous recombination	ko03440	6	0.1827	1.00E+00	sof-miR167b-5p,sof-miR395b-5p,sof-miRN303-5p,sof-miR395c-5p,sof-miR408-3p,sof-miR395a-3p,sof-miR167c-5p
Carbohydrate metabolism	Starch and sucrose metabolism	ko00500	7	0.2055	1.00E+00	sof-miR5384-3p,sof-miRN141-3p,sof-miR319-3p
Endocrine system	Melanogenesis	ko04916	2	0.2114	1.00E+00	sof-miR5384-3p
Circulatory system	Vascular smooth muscle contraction	ko04270	2	0.2215	1.00E+00	sof-miR5384-3p
Amino acid metabolism	Arginine and proline metabolism	ko00330	2	0.2417	1.00E+00	sof-miRN141-3p,sof-miR167c-5p,sof-miR167b-5p
Nervous system	Dopaminergic synapse	ko04728	2	0.2603	1.00E+00	sof-miR5384-3p
Endocrine and metabolic diseases	Insulin resistance	ko04931	2	0.262	1.00E+00	sof-miRN141-3p
Endocrine and metabolic diseases	Non-alcoholic fatty liver disease (NAFLD)	ko04932	2	0.2671	1.00E+00	sof-miRN141-3p

Circulatory system	Adrenergic signaling in cardiomyocytes	ko04261	2	0.2756	1.00E+00	sof-miR5384-3p
Folding, sorting and degradation	RNA degradation	ko03018	8	0.2803	1.00E+00	sof-miRN003-5p,sof-miRN267-5p,sof-miRN145-3p,sof-miR5384-3p,sof-miR408-3p,sof-miR393-3p,sof-miR166g-5p
Nervous system	Long-term potentiation	ko04720	2	0.2992	1.00E+00	sof-miR5384-3p
Signal transduction	Calcium signaling pathway	ko04020	2	0.3026	1.00E+00	sof-miR5384-3p
Endocrine system	GnRH signaling pathway	ko04912	2	0.3094	1.00E+00	sof-miR5384-3p
Translation	RNA transport	ko03013	9	0.3166	1.00E+00	sof-miR156-3p,sof-miR395a-3p,sof-miR166g-5p,sof-miR166e-5p,sof-miRN145-3p,sof-miR395c-5p,sof-miR395b-5p,sof-miR408-3p,sof-miR156-5p
Nervous system	Glutamatergic synapse	ko04724	2	0.3178	1.00E+00	sof-miR5384-3p
Replication and repair	Mismatch repair	ko03430	2	0.384	1.00E+00	sof-miRN303-5p,sof-miR408-3p
Carbohydrate metabolism	Inositol phosphate metabolism	ko00562	2	0.3872	1.00E+00	sof-miRN141-3p,sof-miR166g-5p
Infectious diseases: Bacterial	Vibrio cholerae infection	ko05110	2	0.3872	1.00E+00	sof-miR5384-3p
Translation	Ribosome biogenesis in eukaryotes	ko03008	7	0.4035	1.00E+00	sof-miRN145-3p,sof-miRN267-5p,sof-miR319-3p,sof-miRN003-5p,sof-miR5384-3p,sof-miR166g-5p,sof-miR393-3p
Signal transduction	cAMP signaling pathway	ko04024	2	0.4192	1.00E+00	sof-miR5384-3p
Lipid metabolism	Glycerolipid metabolism	ko00561	2	0.4427	1.00E+00	sof-miR408-3p
Replication and repair	DNA replication	ko03030	2	0.4686	1.00E+00	sof-miRN303-5p,sof-miR408-3p
Folding, sorting and degradation	Proteasome	ko03050	1	0.4797	1.00E+00	sof-miR5384-3p
Replication and repair	Nucleotide excision repair	ko03420	2	0.5446	1.00E+00	sof-miRN303-5p,sof-miR395b-5p,sof-miR395c-5p,sof-miR395a-3p
Signal transduction	Phosphatidylinositol signaling system	ko04070	2	0.5568	1.00E+00	sof-miR166g-5p,sof-miRN141-3p
Xenobiotics biodegradation and metabolism	Polycyclic aromatic hydrocarbon degradation	ko00624	1	0.5907	1.00E+00	sof-miR408-3p
Metabolism of cofactors and vitamins	Ubiquinone and other terpenoid-quinone biosynthesis	ko00130	1	0.6145	1.00E+00	sof-miRN167-3p
Xenobiotics biodegradation and metabolism	Bisphenol degradation	ko00363	1	0.6251	1.00E+00	sof-miR408-3p
Metabolism of terpenoids and polyketides	Limonene and pinene degradation	ko00903	1	0.6319	1.00E+00	sof-miR408-3p
Energy metabolism	Photosynthesis	ko00195	1	0.6404	1.00E+00	sof-miR5384-3p,sof-miRN141-3p
Xenobiotics biodegradation and metabolism	Aminobenzoate degradation	ko00627	1	0.6404	1.00E+00	sof-miR408-3p
Membrane transport	ABC transporters	ko02010	2	0.6456	1.00E+00	sof-miR156-3p,sof-miR5384-3p
Carbohydrate metabolism	Ascorbate and aldarate metabolism	ko00053	1	0.6766	1.00E+00	sof-miR408-3p
Signal transduction	Phospholipase D signaling pathway	ko04072	1	0.6955	1.00E+00	sof-miR166g-5p
Cell motility	Regulation of actin cytoskeleton	ko04810	1	0.7106	1.00E+00	sof-miR166g-5p
Carbohydrate metabolism	Pyruvate metabolism	ko00620	1	0.7275	1.00E+00	sof-miRN245-5p
Lipid metabolism	Glycerophospholipid metabolism	ko00564	2	0.7381	1.00E+00	sof-miR408-3p
Infectious diseases: Parasitic	Toxoplasmosis	ko05145	4	0.7558	1.00E+00	sof-miRN141-3p,sof-miR166e-5p
Carbohydrate metabolism	Pentose and glucuronate interconversions	ko00040	1	0.7584	1.00E+00	sof-miRN141-3p
Folding, sorting and degradation	Protein processing in endoplasmic reticulum	ko04141	3	0.7656	1.00E+00	sof-miRN014-3p
Signal transduction	FoxO signaling pathway	ko04068	2	0.7738	1.00E+00	sof-miRN141-3p
Carbohydrate metabolism	Glyoxylate and dicarboxylate metabolism	ko00630	1	0.7788	1.00E+00	sof-miR5384-3p
Biosynthesis of other secondary metabolites	Flavonoid biosynthesis	ko00941	1	0.8205	1.00E+00	sof-miR5384-3p
Metabolism of other amino acids	Cyanoamino acid metabolism	ko00460	1	0.8205	1.00E+00	sof-miRN003-5p
Metabolism of terpenoids and polyketides	Terpenoid backbone biosynthesis	ko00900	1	0.8262	1.00E+00	sof-miRN141-3p
Biosynthesis of other secondary metabolites	Stilbenoid, diarylheptanoid and gingerol biosynthesis	ko00945	1	0.8286	1.00E+00	sof-miR408-3p
Infectious diseases: Viral	HTLV-I infection	ko05166	2	0.8426	1.00E+00	sof-miR5384-3p
Transport and catabolism	Peroxisome	ko04146	1	0.8438	1.00E+00	sof-miR5384-3p
Cell growth and death	Apoptosis	ko04210	4	0.8473	1.00E+00	sof-miR5384-3p,sof-miR166e-5p
Infectious diseases: Viral	Measles	ko05162	3	0.864	1.00E+00	sof-miR395a-3p,sof-miR166e-5p,sof-miR395c-5p,sof-miR395b-5p
Translation	mRNA surveillance pathway	ko03015	2	0.8762	1.00E+00	sof-miR166g-5p,sof-miRN145-3p
Transport and catabolism	Lysosome	ko04142	1	0.8808	1.00E+00	sof-miRN245-5p
Transport and catabolism	Endocytosis	ko04144	1	0.8862	1.00E+00	sof-miR166g-5p
Infectious diseases: Viral	Epstein-Barr virus infection	ko05169	4	0.8979	1.00E+00	sof-miRN245-5p,sof-miR5384-3p,sof-miRN003-5p

Biosynthesis of other secondary metabolites	Phenylpropanoid biosynthesis	ko00940	2	0.9019	1.00E+00	sof-miR5384-3p
Infectious diseases: Parasitic	Leishmaniasis	ko05140	2	0.9147	1.00E+00	sof-miR166e-5p
Carbohydrate metabolism	Amino sugar and nucleotide sugar metabolism	ko00520	1	0.9152	1.00E+00	sof-miR5384-3p
Infectious diseases: Parasitic	Chagas disease (American trypanosomiasis)	ko05142	2	0.9316	1.00E+00	sof-miR166e-5p
Infectious diseases: Bacterial	Pertussis	ko05133	2	0.9446	1.00E+00	sof-miR166e-5p
Transcription	RNA polymerase	ko03020	2	0.9465	1.00E+00	sof-miRN003-5p,sof-miRN245-5p
Global and overview maps	Metabolic pathways	ko01100	38	0.9513	1.00E+00	sof-miR395a-3p,sof-miR319-3p,sof-miR167c-5p,sof-miRN141-3p,sof-miRN003-5p,sof-miRN245-5p,sof-miR166g-5p,sof-miR167b-5p,sof-miR167a-3p,sof-miR395b-5p,sof-miR395c-5p,sof-miR5384-3p,sof-miR393-5p,sof-miRN167-3p,sof-miR408-3p,sof-miR393-3p
Nervous system	Neurotrophin signaling pathway	ko04722	2	0.955	1.00E+00	sof-miR166e-5p
Folding, sorting and degradation	Ubiquitin mediated proteolysis	ko04120	1	0.9565	1.00E+00	sof-miR396c-3p,sof-miRN375-3p,sof-miR396a-5p,sof-miR396b-5p
Replication and repair	Fanconi anemia pathway	ko03460	1	0.9683	1.00E+00	sof-miRN303-5p
Infectious diseases: Viral	Influenza A	ko05164	2	0.9701	1.00E+00	sof-miR166e-5p
Infectious diseases: Bacterial	Tuberculosis	ko05152	2	0.9786	1.00E+00	sof-miR166e-5p
Nucleotide metabolism	Pyrimidine metabolism	ko00240	2	0.989	1.00E+00	sof-miRN245-5p,sof-miRN003-5p
Global and overview maps	Microbial metabolism in diverse environments	ko01120	3	0.9918	1.00E+00	sof-miR5384-3p,sof-miR408-3p,sof-miRN141-3p
Nucleotide metabolism	Purine metabolism	ko00230	2	0.9921	1.00E+00	sof-miRN245-5p,sof-miRN003-5p
Global and overview maps	Carbon metabolism	ko01200	1	0.9935	1.00E+00	sof-miR5384-3p
Global and overview maps	Biosynthesis of secondary metabolites	ko01110	17	0.9961	1.00E+00	sof-miRN003-5p,sof-miR395c-5p,sof-miR395b-5p,sof-miR395a-3p,sof-miR408-3p,sof-miRN167-3p,sof-miR5384-3p
Global and overview maps	Biosynthesis of antibiotics	ko01130	2	0.9994	1.00E+00	sof-miR5384-3p,sof-miRN141-3p

Table S3. SNPs in sugarcane miRNAs.

miRNA	Position	Type	B1		B2		Q1		Q2		T1		T2	
			miRNP read	Percent	miRNP read	Percent	miRNP read	Percent	miRNP read	Percent	miRNP read	Percent	miRNP read	Percent
sof-miR156-3p	5	A->G	7	63.64%	6	37.50%	33	32.35%	0	0	0	0	0	0
sof-miR156-5p	15	T->C	90	18.07%	150	5.47%	694	4.32%	178	19.80%	90	9.32%	7	3.55%
sof-miR166a-3p	12	T->C	660	0.04%	392	0.05%	263	0.04%	13032	1.89%	38645	4.82%	535	0.11%
sof-miR166b-5p	8	T->G	265	53.54%	145	22.45%	0	0	259	48.41%	73	17.06%	42	64.62%
sof-miR166b-5p	12	G->A	5	1.01%	5	0.77%	0	0	0	0	25	5.84%	0	0
sof-miR166c-3p	15	T->C	685	0.04%	413	0.04%	334	0.04%	12861	1.81%	38092	4.36%	542	0.11%
sof-miR166d-3p	12	T->C	681	0.04%	410	0.04%	332	0.04%	12860	1.82%	38086	4.42%	537	0.11%
sof-miR166f-3p	12	T->C	729	0.04%	580	0.05%	359	0.04%	13131	1.57%	38747	3.94%	544	0.11%
sof-miR166g-3p	16	A->T	372	0.94%	263	1.14%	219	0.90%	294	0.90%	330	1.79%	171	1.32%
sof-miR172-5p	9	A->G	6	8.00%	78	50.65%	15	34.09%	0	0	40	31.25%	0	0
sof-miR2275-5p	8	G->T	0	0	0	0	0	0	0	0	27	27.55%	13	39.39%
sof-miR395a-3p	8	T->C	0	0	11	3.57%	0	0	0	0	0	0	0	0
sof-miR395a-3p	13	G->A	46	56.79%	130	42.21%	13	14.13%	7	25.93%	299	71.36%	14	43.75%
sof-miR395a-3p	14	G->T	12	14.81%	26	8.44%	12	13.04%	0	0	16	3.82%	0	0
sof-miR395a-5p	12	A->G	0	0	0	0	0	0	0	0	13	41.94%	0	0
sof-miR395b-3p	8	T->G	0	0	0	0	0	0	0	0	13	65.00%	0	0
sof-miR395b-5p	8	T->C	0	0	11	3.57%	0	0	0	0	0	0	0	0
sof-miR395b-5p	13	G->A	46	56.79%	130	42.21%	13	14.44%	7	25.93%	299	73.65%	14	43.75%
sof-miR395b-5p	14	G->T	12	14.81%	26	8.44%	12	13.33%	0	0	16	3.94%	0	0
sof-miR395c-5p	8	T->C	0	0	11	3.57%	0	0	0	0	0	0	0	0
sof-miR395c-5p	13	G->A	46	56.79%	130	42.21%	13	14.13%	7	25.93%	299	71.36%	14	46.67%
sof-miR395c-5p	14	G->T	12	14.81%	26	8.44%	12	13.04%	0	0	16	3.82%	0	0
sof-miR396b-3p	15	A->T	0	0	0	0	15	40.54%	0	0	0	0	0	0
sof-miR5564a-3p	15	G->C	0	0	0	0	0	0	51	68.00%	32	66.67%	12	52.17%
sof-miR5564b-3p	10	A->T	1035	85.05%	1798	93.99%	0	0	0	0	764	91.50%	283	83.48%
sof-miR5564b-3p	16	T->G	80	6.57%	73	3.82%	43	27.04%	96	45.93%	39	4.67%	10	2.95%
sof-miR5568b-5p	6	A->G	5	2.44%	0	0	19	4.97%	12	3.68%	7	1.51%	0	0
sof-miR5568b-5p	10	G->T	34	16.59%	156	36.11%	64	16.75%	31	9.51%	124	26.78%	9	12.33%
sof-miR5568b-5p	11	A->T	0	0	15	3.47%	9	2.36%	9	2.76%	0	0	0	0
sof-miR5568b-5p	12	C->T	0	0	9	2.08%	8	2.09%	20	6.14%	22	4.75%	7	9.59%
sof-miR5568d-3p	6	T->C	10	25.00%	0	0	21	28.38%	0	0	0	0	9	64.29%
sof-miR5568d-3p	14	A->G	0	0	0	0	19	25.68%	15	34.88%	0	0	0	0
sof-miR5568e-5p	6	A->G	16	6.72%	6	2.41%	17	6.75%	24	6.33%	6	1.53%	0	0
sof-miR5568e-5p	10	G->T	8	3.36%	37	14.86%	17	6.75%	14	3.69%	42	10.71%	0	0
sof-miR5568e-5p	11	A->T	93	39.08%	48	19.28%	45	17.86%	131	34.56%	113	28.83%	25	33.78%
sof-miR5568e-5p	12	C->T	7	2.94%	11	4.42%	8	3.17%	23	6.07%	24	6.12%	0	0
sof-miRN001-5p	5	C->A	69	98.57%	97	100.00%	46	100.00%	60	100.00%	40	93.02%	28	96.55%
sof-miRN003-3p	8	G->A	129	100.00%	0	0	0	0	0	0	0	0	0	0
sof-miRN003-5p	12	C->A	31	33.33%	27	61.36%	15	83.33%	12	66.67%	20	90.91%	0	0
sof-miRN004-3p	18	A->G	9	42.86%	7	36.84%	12	57.14%	12	46.15%	13	37.14%	9	69.23%
sof-miRN006-5p	16	G->A	55	94.83%	39	100.00%	45	88.24%	58	100.00%	28	100.00%	29	96.67%
sof-miRN007-5p	5	A->G	5	2.44%	0	0	19	4.97%	12	3.68%	7	1.51%	0	0
sof-miRN007-5p	9	G->T	34	16.59%	156	36.11%	64	16.75%	31	9.51%	124	26.78%	9	12.33%
sof-miRN007-5p	10	A->T	0	0	15	3.47%	9	2.36%	9	2.76%	0	0	0	0
sof-miRN007-5p	11	C->T	0	0	9	2.08%	8	2.09%	20	6.14%	22	4.75%	7	9.59%
sof-miRN008-3p	11	A->T	11	100.00%	5	62.50%	9	100.00%	0	0	0	0	0	0
sof-miRN009-5p	6	T->C	57	98.28%	66	86.84%	51	100.00%	92	93.88%	65	97.01%	58	98.31%
sof-miRN010-5p	5	G->A	9	13.64%	25	24.75%	26	13.13%	13	7.69%	21	18.58%	5	15.15%
sof-miRN014-3p	2	A->G	0	0	146	97.33%	802	90.83%	338	88.02%	412	84.43%	321	98.47%
sof-miRN020-3p	15	G->T	73	70.19%	23	69.70%	82	89.13%	98	73.68%	93	83.78%	239	80.74%
sof-miRN027-5p	9	G->T	27	25.47%	122	45.52%	52	26.80%	17	13.08%	89	38.70%	5	18.52%
sof-miRN027-5p	10	A->T	0	0	15	5.60%	9	4.64%	9	6.92%	0	0	0	0
sof-miRN028-3p	10	T->C	0	0	0	0	12	13.19%	0	0	0	0	0	0
sof-miRN030-5p	9	T->C	0	0	0	0	0	0	12	80.00%	0	0	0	0
sof-miRN043-5p	16	C->T	7	58.33%	0	0	5	100.00%	11	57.89%	6	100.00%	6	54.55%
sof-miRN057-5p	5	G->C	0	0	11	15.28%	5	1.43%	0	0	0	0	0	0
sof-miRN057-5p	5	G->T	0	0	16	22.22%	60	17.19%	0	0	8	10.53%	0	0
sof-miRN057-5p	9	C->T	0	0	0	0	35	10.03%	0	0	9	11.84%	0	0
sof-miRN057-5p	10	C->T	0	0	0	0	7	2.01%	16	18.18%	5	6.58%	0	0
sof-miRN057-5p	12	A->G	20	26.32%	0	0	16	4.58%	7	7.95%	10	13.16%	37	50.68%
sof-miRN059-3p	5	T->C	0	0	0	0	10	50.00%	0	0	14	45.16%	0	0
sof-miRN059-3p	17	A->G	14	93.33%	9	90.00%	10	50.00%	8	100.00%	17	54.84%	7	100.00%
sof-miRN061-5p	13	T->A	0	0	0	0	83	71.55%	0	0	0	0	0	0
sof-miRN078-3p	13	G->A	8	50.00%	10	76.92%	0	0	20	83.33%	15	100.00%	0	0
sof-miRN083-3p	8	T->G	27	71.05%	32	72.73%	45	72.58%	23	79.31%	42	93.33%	11	91.67%
sof-miRN092-3p	8	G->A	14	35.00%	7	25.93%	0	0	6	25.00%	13	29.55%	9	22.50%
sof-miRN095-5p	16	T->A	12	85.71%	0	0	25	80.65%	21	80.77%	13	81.25%	23	85.19%
sof-miRN097-5p	9	T->C	0	0	0	0	0	0	0	0	13	12.62%	0	0
sof-miRN099-5p	7	C->T	25	12.56%	9	6.29%	23	7.08%	14	6.64%	42	16.54%	10	10.53%
sof-miRN099-5p	15	G->A	10	5.03%	7	4.90%	15	4.62%	0	0	0	0	0	0
sof-miRN108-3p	8	C->T	14	16.28%	16	47.06%	7	6.48%	13	23.21%	14	20.90%	14	23.33%
sof-miRN108-3p	9	C->T	11	12.79%	7	20.59%	24	22.22%	5	8.93%	15	22.39%	9	15.00%
sof-miRN108-3p	12	G->A	18	20.93%	0	0	7	6.48%	14	25.00%	11	16.42%	5	8.33%
sof-miRN109-5p	7	G->A	100	54.95%	67	58.77%	58	50.43%	141	55.29%	117	60.62%	26	59.09%
sof-miRN109-5p	12	T->A	9	4.95%	0	0	0	0	14	5.49%	0	0	0	0
sof-miRN109-5p	17	T->C	39	21.43%	29	25.44%	41	35.65%	35	13.73%	29	15.03%	14	31.82%
sof-miRN113-3p	6	T->C	66	78.57%	44	95.65%	373	94.91%	136	95.77%	122	87.14%	92	88.46%
sof-miRN115-5p	16	C->T	15	11.45%	15	15.31%	62	22.79%	7	7.87%	21	15.56%	31	28.97%
sof-miRN145-3p	18	C->T	14	46.67%	0	0	0	0	0	0	5	4.46%	0	0
sof-miRN154-5p	7	G->A	39	25.49%	12	11.01%	10	3.23%	5	3.40%	29	12.72%	11	8.03%
sof-miRN155-5p	7	G->A	39	25.49%	12	11.01%	10	3.23%	5	3.40%	29	12.72%	11	8.03%
sof-miRN158-3p	16	A->G	12	100.00%	0	0	6	100.00%	6	85.71%	7	100.00%	14	100.00%
sof-miRN168-5p	14	G->A	0	0	0	0	156	100.00%	0	0	0	0	0	0
sof-miRN173-5p	9	A->G	17	100.00%	11	100.00%	26	100.00%	5	100.00%	0	0	10	100.00%
sof-miRN177-3p	5	A->G	0	0	14	7.14%	13	12.50%	0	0	0	0	0	0
sof-miRN177-3p	19	C->T	49	76.56%	148	75.51%	59	56.73%	58	76.32%	60	89.55%	112	96.55%
sof-miRN184-3p	10	G->A	0	0	13	7.18%	15	3.47%	0	0	0	0	0	0
sof-miRN192-3p	7	T->A	0	0	0	0	15	68.18%	0	0	0	0	0	0
sof-miRN192-3p	11	C->T	0	0	0	0	0	0	12	70.59%	0	0	0	0
sof-miRN196-5p	12	C->T	0	0	22	75.86%	12	42.86%	9	56.25%	10	52.63%	12	40.00%
sof-miRN201-3p	13	A->G	17	6.61%	19	5.04%	24	3.80%	40	3.96%	29	8.48%	9	2.59%
sof-miRN201-3p	19	T->G	9	3.50%	6	1.59%	66	10.46%	68	6.73%	0	0	6	1.72%
sof-miRN205-5p	8	T->A	591	98.50%	597	99.01%	77	100.00%	157	99.37%	442	99.55%	97	100.00%
sof-miRN207-5p	17	C->T	10	10.64%	0	0	0	0	12	5.63%	0	0	11	5.58%
sof-miRN210-5p	13	C->T	28	84.85%	19	90.48%	5	55.56%	29	80.56%	14	93.33%	23	85.19%
sof-miRN219-5p	5	G->A	48	100.00%	22	100.00%	8	100.00%	12	100.00%	7	100.00%	16	100.00%
sof-miRN222-5p	9	C->T	10	24.39%	7	9.09%	0	0	6	8.11%	15	24.19%	0	0
sof-miRN233-3p	7	C->T	437	100.00%	0	0	0	0	0	0	0	0	0	0
sof-miRN233-5p	12	C->A	32	35.56%	27	61.36%	15	83.33%	12	57.14%	20	90.91%	0	0
sof-miRN238-3p	16	C->T	10	45.45%	12	44.44%	0	0	0	0	0	0	0	0
sof-miRN246-3p	13	G->A	6	21.43%	0	0	9	64.29%	7	25.93%	6	42.86%	13	59.09%
sof-miRN251-3p	9	G->A	13	92.86%	12	85.71%	0	0	0	0	5	100.00%	0	0
sof-miRN271-3p	4	C->T	0	0	0	0	0	0	11	57.89%	0	0	0	0
sof-miRN281-3p	6	G->A	7	100.00%	54	100.00%	43	100.00%	8	100.00%	26	100.00%	24	100.00%
sof-miRN296-5p	20	C->T	12	54.55%	0	0	0	0	0	0	0	0	9	90.00%
sof-miRN301-5p	12	C->A	17	100.00%	11	47.83%	0	0	12	70.59%	7	87.50%	10	100.00%
sof-miRN302-3p	20	T->A	21	95.45%	8	80.00%	7	100.00%	0	0	7	100.00%	11	91.67%
sof-miRN303-5p	7	T->C	59	83.10%	49	74.24%	55	88.71%	66	98.51%	180	86.96%	40	22.10%
sof-miRN310-3p	14	G->A	0	0	5	16.67%	0	0	0	0	0	0	12	9.09%
sof-miRN310-3p	18	A->G	55	71.43%	16	53.33%	48	94.12%	61	74.39%	36	80.00%	89	67.42%
sof-miRN322-5p	18	C->T	0	0	0	0	0	0	14	58.33%	0	0	0	0
sof-miRN324-5p	11	G->A	11	50.00%	0	0	13	30.23%	0	0	0	0	0	0
sof-miRN325-5p	9	G->A	0	0	0	0	22	17.19%	0	0	0	0	0	0
sof-miRN330-3p	13	T->A	0	0	0	0	35	52.24%	0	0	0	0	0	0
sof-miRN339-5p	13	C->T	5	10.00%	0	0	0	0	30	16.30%	0	0	0	0
sof-miRN341-5p	9	T->A	241	100.00%	153	100.00%	168	100.00%	295	100.00%	169	100.00%	39	100.00%
sof-miRN343-3p	4	C->T	20	0.53%	0	0	14	0.78%	62	0.44%	76	1.10%	0	0
sof-miRN366-3p	9	G->A	11	11.00%	17	20.00%	24	15.79%	21	28.38%	14	17.50%	11	8.33%
sof-miRN366-3p	9	G->T	9	9.00%	7	8.24%	0	0	0	0	6	7.50%	11	8.33%
sof-miRN369-3p	18	T->G	43	74.14%	7	58.33%	99	95.19%	42	56.76%	28	53.85%	7	36.84%

sof-miRN371-5p	8	C->A	49	98.00%	29	82.86%	20	71.43%	111	100.00%	50	90.91%	25	100.00%
sof-miRN379-5p	17	C->T	0	0	0	0	142	9.02%	0	0	0	0	0	0
sof-miRN391-3p	14	A->C	9	75.00%	8	72.73%	0	0	12	80.00%	7	87.50%	7	58.33%
sof-miRN392-3p	13	G->A	0	0	0	0	0	0	14	100.00%	0	0	0	0
sof-miRN396-3p	6	C->T	2249	53.02%	2364	58.18%	5713	69.38%	958	29.12%	1281	33.65%	1567	47.64%
sof-miRN396-3p	7	G->A	275	6.48%	249	6.13%	443	5.38%	319	9.70%	245	6.44%	196	5.96%
sof-miRN396-3p	11	C->A	70	1.65%	41	1.01%	32	0.39%	89	2.71%	69	1.81%	100	3.04%
sof-miRN396-3p	13	T->A	64	1.51%	29	0.71%	78	0.95%	58	1.76%	110	2.89%	22	0.67%
sof-miRN396-3p	16	G->A	210	4.95%	207	5.09%	543	6.59%	204	6.20%	121	3.18%	120	3.65%
sof-miRN401-3p	15	A->G	0	0	0	0	18	100.00%	0	0	6	66.67%	16	100.00%
sof-miRN402-3p	4	C->T	18	81.82%	0	0	0	0	0	0	0	0	0	0
sof-miRN405-5p	8	G->A	12	38.71%	0	0	8	26.67%	8	10.67%	7	14.00%	0	0
sof-miRN405-5p	19	G->A	0	0	0	0	0	0	13	17.33%	0	0	0	0
sof-miRN406-5p	9	G->C	11	91.67%	9	69.23%	0	0	11	45.83%	0	0	7	77.78%
sof-miRN407-3p	15	A->T	9	100.00%	9	100.00%	24	100.00%	0	0	14	100.00%	0	0

Table S4. RT primers, forward and reverse primers for cDNA synthesize and RT-qPCR.

Target	RT primer	forward primer	reverse primer
sof-miR159-3p	GTCGTATCCAGTGCGTGTCGTGGAGTCGGCAATTGCACTGGATACGACAGGCGA	TTCGGGTACCATTCTGATC	TGCGTGTCGTGGAGTCG
sof-miR166a-3p	GTCGTATCCAGTGCGTGTCGTGGAGTCGGCAATTGCACTGGATACGACGGGGAA	TCGGACCAGGCTTCATTC	
sof-miR396a-5p	GTCGTATCCAGTGCGTGTCGTGGAGTCGGCAATTGCACTGGATACGACCAGTTC	TCCACAGGCTTTCTTGAAC	
sof-5564b-3p	GTCGTATCCAGTGCGTGTCGTGGAGTCGGCAATTGCACTGGATACGACGGAGGA	AGCTGTTCGACGAATTCCT	
sof-miR5564bV-3p	GTCGTATCCAGTGCGTGTCGTGGAGTCGGCAATTGCACTGGATACGACGGAATT	TccagctgttcgTcgaatt	
actin		TACGTTGCGATTCAAGCCGT	CCAAACGAAGAATGGCATGG

Fig. S1

A

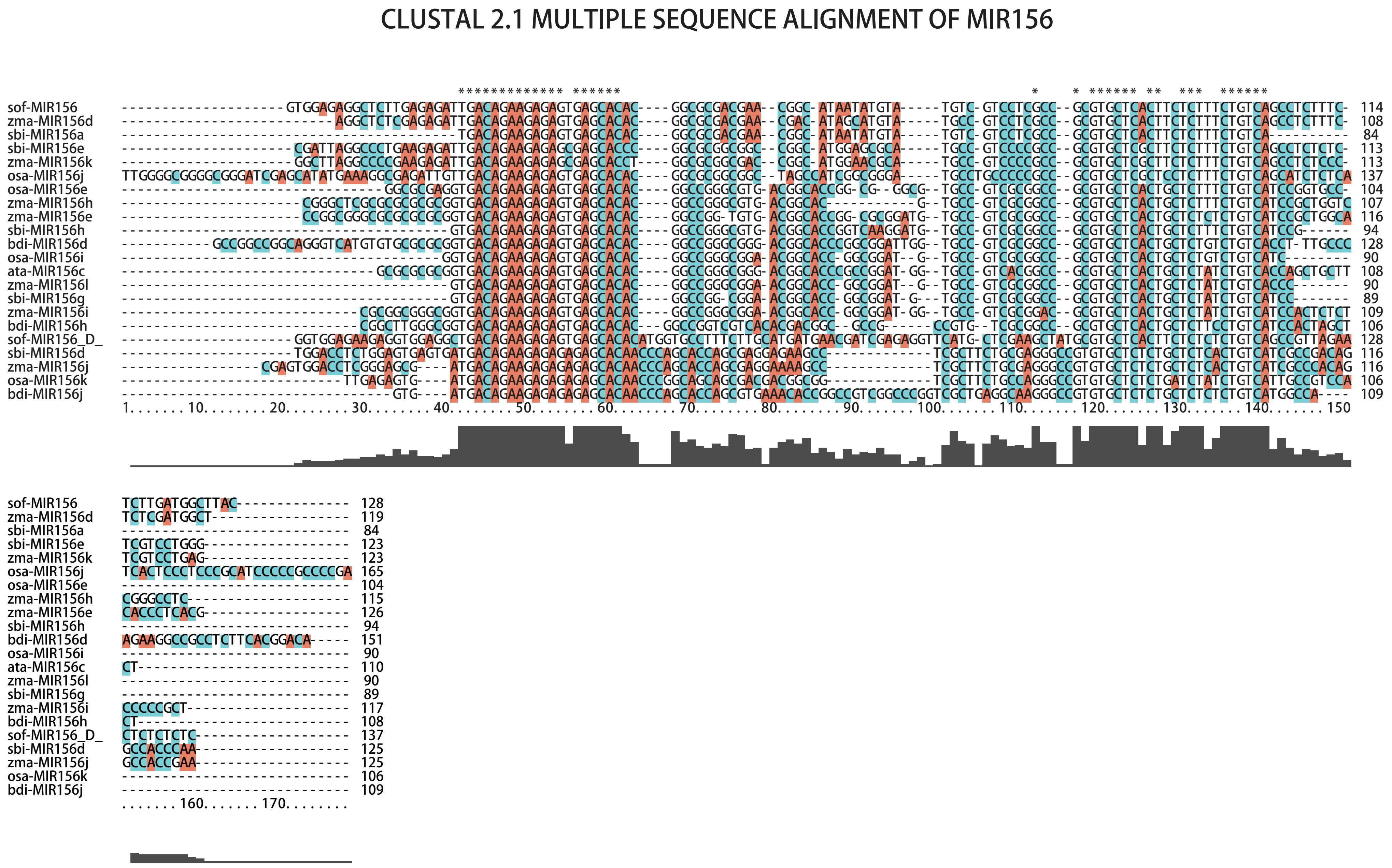

CLUSTAL 2.1 MULTIPLE SEQUENCE ALIGNMENT OF MIR156
sof-MIR156
zma-MIR156d
sbi-MIR156a
sbi-MIR156e
zma-MIR156k
osa-MIR156j
osa-MIR156e
zma-MIR156h
zma-MIR156e
sbi-MIR156h
bdi-MIR156d
osa-MIR156i
ata-MIR156c
zma-MIR156l
sbi-MIR156g
zma-MIR156i
bdi-MIR156h
sof-MIR156_D_
sbi-MIR156d
zma-MIR156j
osa-MIR156k
bdi-MIR156j

B

CLUSTAL 2.1 MULTIPLE SEQUENCE ALIGNMENT OF MIR160

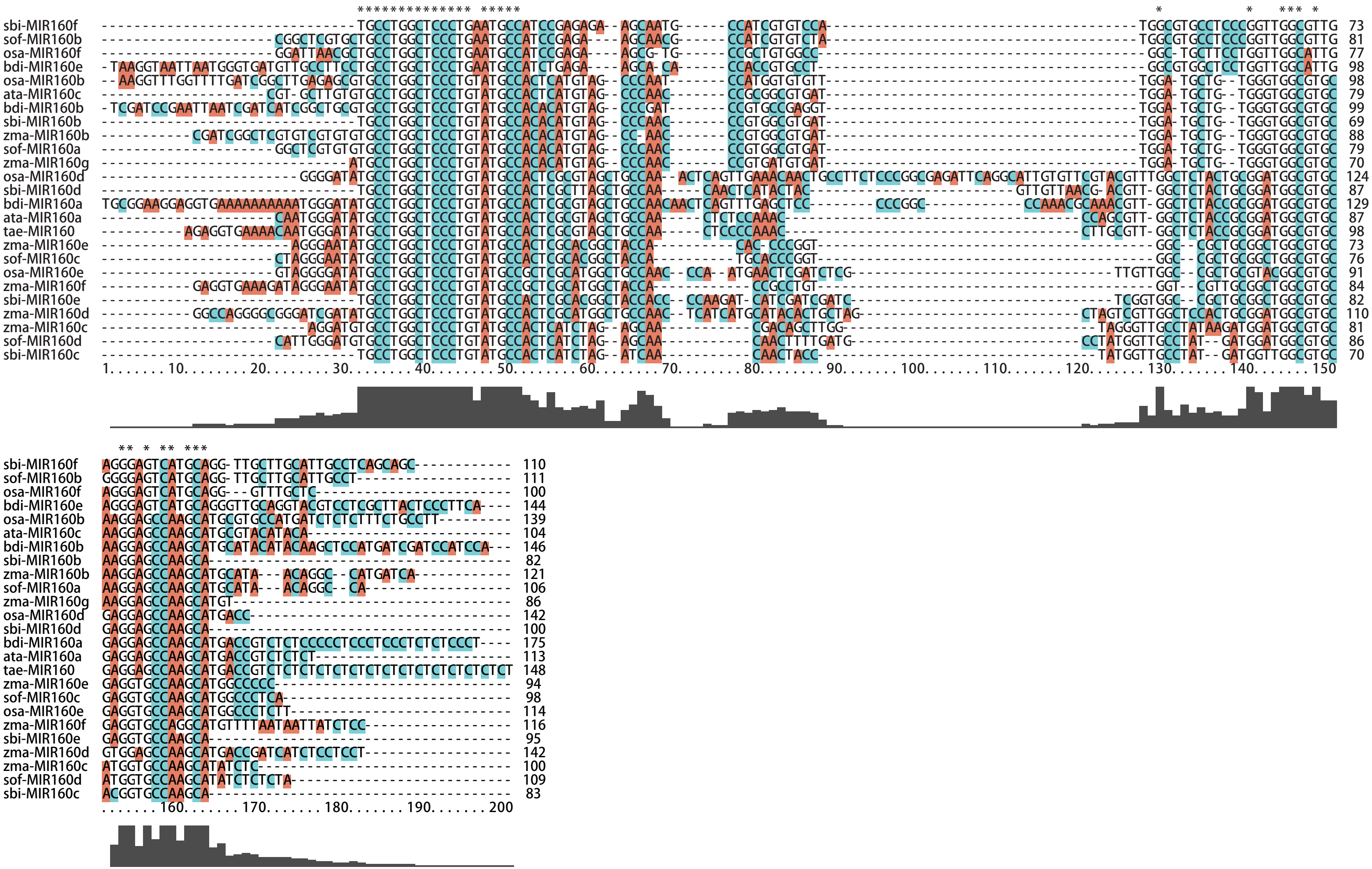

C

CLUSTAL 2.1 MULTIPLE SEQUENCE ALIGNMENT OF MIR166

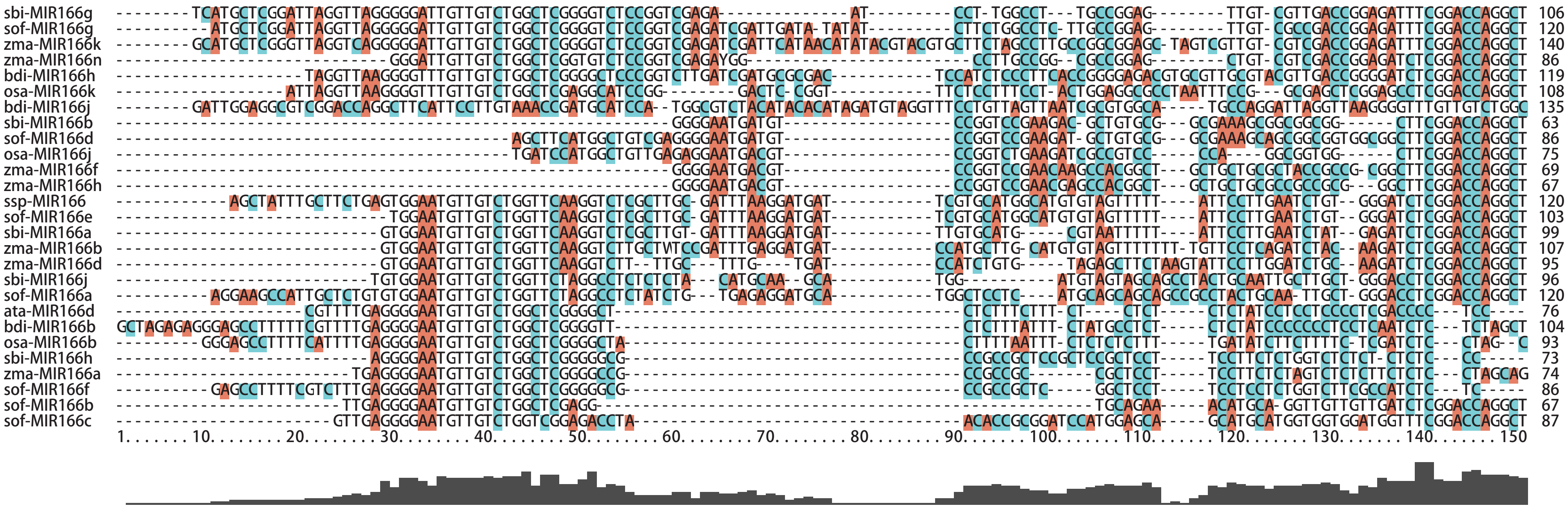

D

CLUSTAL 2.1 MULTIPLE SEQUENCE ALIGNMENT OF MIR167

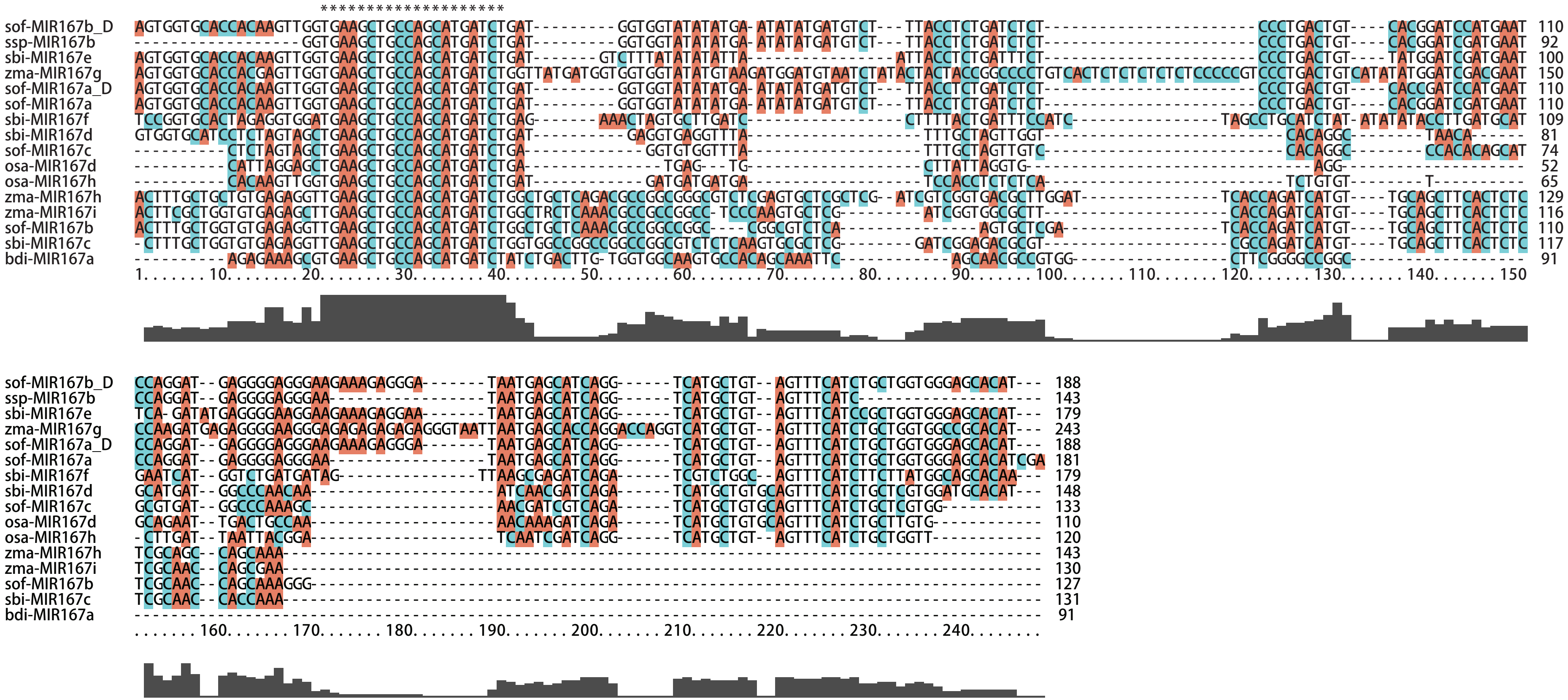

E

CLUSTAL 2.1 MULTIPLE SEQUENCE ALIGNMENT OF MIR171

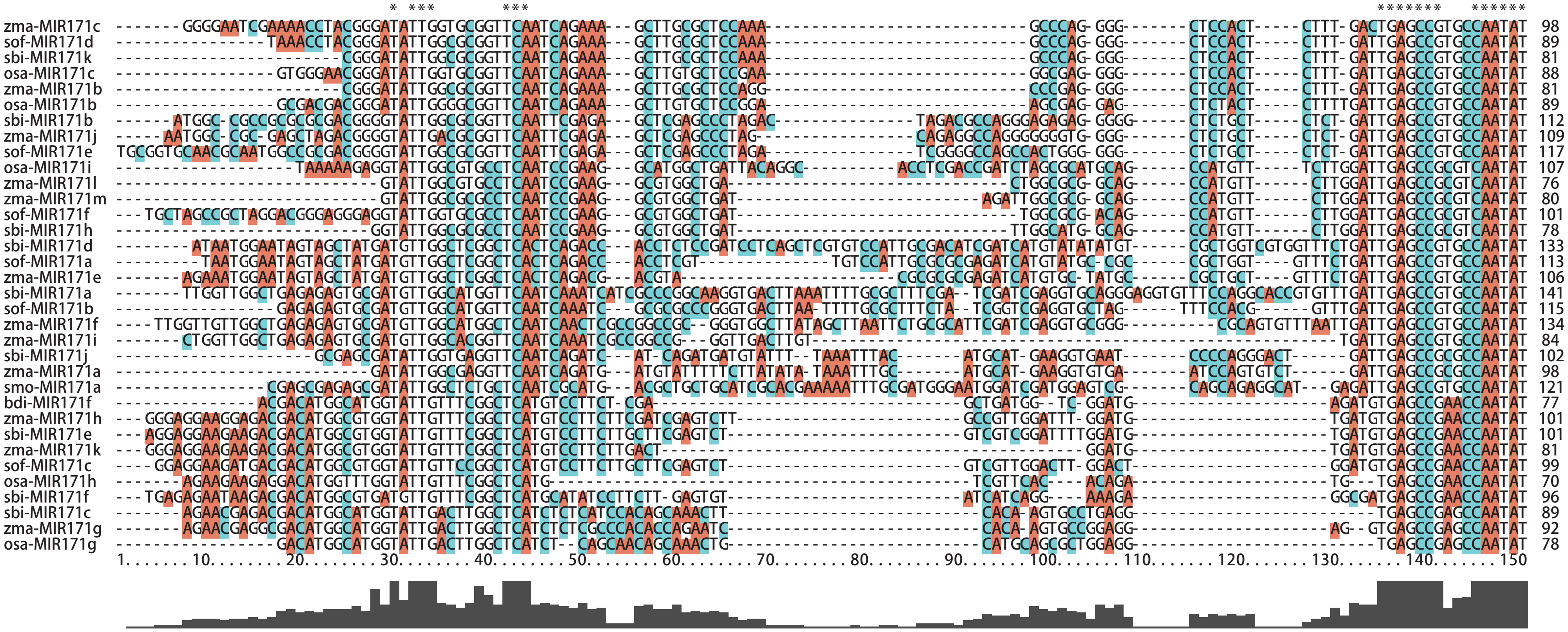

F

CLUSTAL 2.1 MULTIPLE SEQUENCE ALIGNMENT OF MIR396

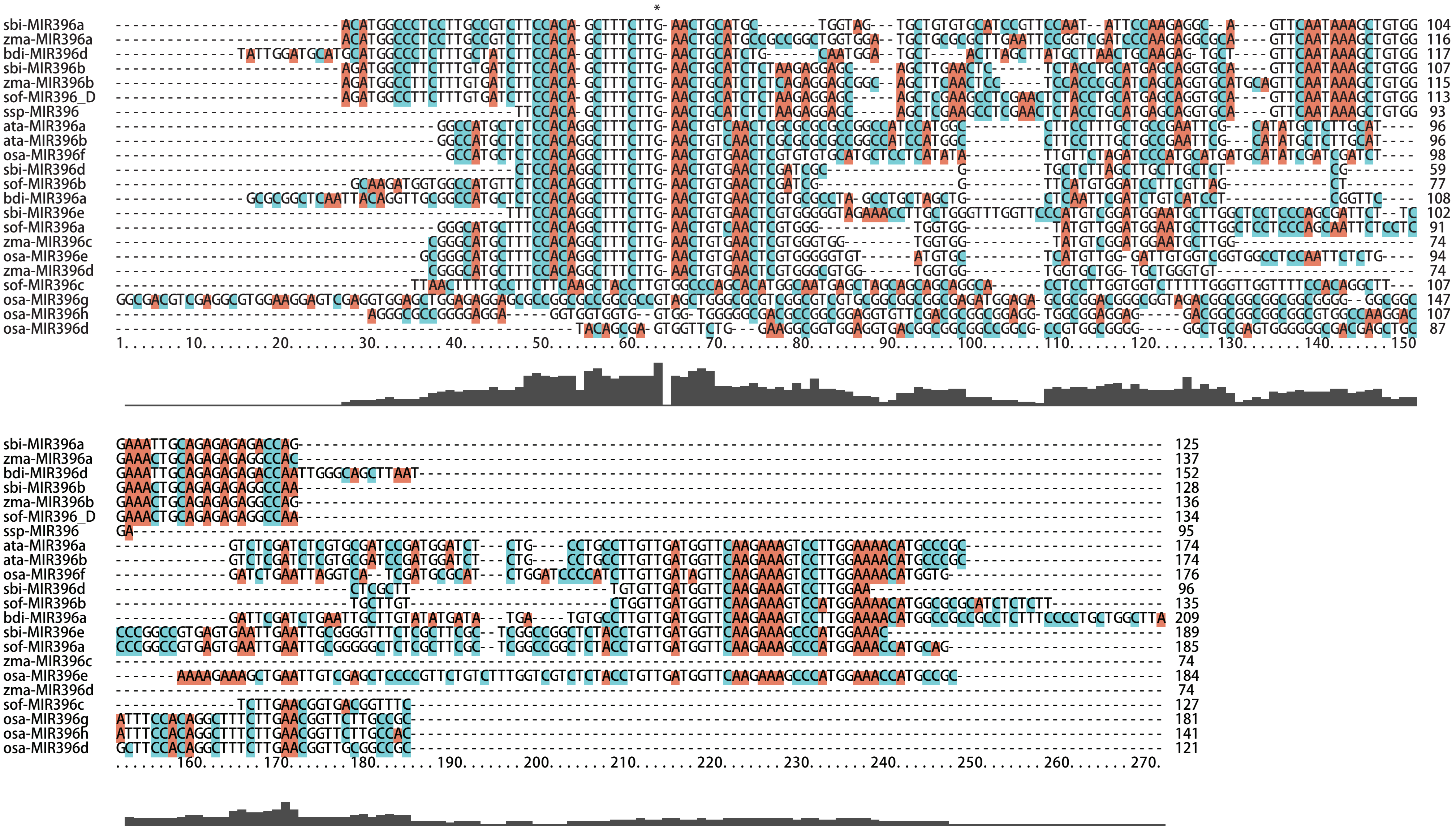

Supplement: Supplementary file 2 — Summary of supplementary tables and figures. (PDF 1132 kb) [file 12864_2017_4053_MOESM2_ESM.pdf]
